# Allocation of distinct organ fates from a precursor field requires a shift in expression and function of gene regulatory networks

**DOI:** 10.1371/journal.pgen.1007185

**Published:** 2018-01-19

**Authors:** Sneha Palliyil, Jinjin Zhu, Luke R. Baker, Sarah D. Neuman, Arash Bashirullah, Justin P. Kumar

**Affiliations:** 1 Department of Biology, Indiana University, Bloomington, Indiana, United States of America; 2 Division of Pharmaceutical Sciences, University of Wisconsin, Madison, Wisconsin, United States of America; Washington University in Saint Louis School of Medicine, UNITED STATES

## Abstract

A common occurrence in metazoan development is the rise of multiple tissues/organs from a single uniform precursor field. One example is the anterior forebrain of vertebrates, which produces the eyes, hypothalamus, diencephalon, and telencephalon. Another instance is the *Drosophila* wing disc, which generates the adult wing blade, the hinge, and the thorax. Gene regulatory networks (GRNs) that are comprised of signaling pathways and batteries of transcription factors parcel the undifferentiated field into discrete territories. This simple model is challenged by two observations. First, many GRN members that are thought to control the fate of one organ are actually expressed throughout the entire precursor field at earlier points in development. Second, each GRN can simultaneously promote one of the possible fates choices while repressing the other alternatives. It is therefore unclear how GRNs function to allocate tissue fates if their members are uniformly expressed and competing with each other within the same populations of cells. We address this paradigm by studying fate specification in the *Drosophila* eye-antennal disc. The disc, which begins its development as a homogeneous precursor field, produces a number of adult structures including the compound eyes, the ocelli, the antennae, the maxillary palps, and the surrounding head epidermis. Several selector genes that control the fates of the eye and antenna, respectively, are first expressed throughout the entire eye-antennal disc. We show that during early stages, these genes are tasked with promoting the growth of the entire field. Upon segregation to distinct territories within the disc, each GRN continues to promote growth while taking on the additional roles of promoting distinct primary fates and repressing alternate fates. The timing of both expression pattern restriction and expansion of functional duties is an elemental requirement for allocating fates within a single field.

## Author summary

A battery of transcription factors collectively called the retinal determination (RD) network controls the earliest steps in the specification of the fruit fly compound eye. Loss-of-function mutations lead to the loss of the compound eyes while over-expression of RD network members in non-retinal tissues induces the formation of ectopic eyes. These observations suggest that the network governs the growth, specification, and patterning of the eye field. Recent studies have also shown that the RD network represses the fates of the non-ocular tissues that are also derived from the disc such as the antenna, maxillary palp, and head epidermis. One inconsistency in the model for how this network controls eye specification is that many of its members are expressed throughout the entire eye-antennal disc. In this study, we show that early in development, the RD network is expressed throughout and promotes the growth of the entire eye-antennal disc. After the initial growth phase, the expression of these genes is restricted to just the eye field. This temporal and spatial limiting of the RD network to the developing eye is essential so that its role can expand to include promoting eye specification and repressing non-ocular fates.

## Introduction

In organisms as diverse as flies and humans, it is common for multiple adult tissues/organs to originate from adjacent territories within a uniform precursor field. For example, the developing anterior forebrain in vertebrates gives rise to the eyes, hypothalamus, diencephalon, and telencephalon [1,2]. Likewise, the developing wing disc of *Drosophila* produces the adult wing, hinge, and thorax [3]. In these instances, it has been shown that gene regulatory networks (GRNs), consisting of signaling pathways and transcription factors, subdivide the precursor field and then separately specify the fate of the distinct territories that are contained therein. The GRNs that are involved in this process are thought to promote growth and specification of each tissue while acting to block tissues from adopting the wrong fate. This ensures that each organ reaches the appropriate size, adopts the correct fate, is positioned properly within the body plan, and has all the requisite cell types. This simple and straightforward model for establishing multiple fates from a single homogeneous precursor is at odds with observations that many GRN members, including master control genes, are expressed broadly within the precursor field. It is difficult to envision how GRNs function to allocate tissue fates if their members are uniformly expressed and competing with each other within the same populations of cells. We have examined the role that the retinal determination (RD) network plays in the *Drosophila* eye-antennal disc, and our results provide a mechanistic solution to this unresolved conflict.

During *Drosophila* embryogenesis, several different populations of cells coalesce to form the eye-antennal disc [4]. This monolayer epithelium, which initially is uniform, eventually gives rise to a number of head structures, including the visual system (compound eyes and ocelli), the olfactory system (antennae and maxillary palps), and the surrounding head epidermis. During the earliest stages of development, the eye-antennal disc does not have any obvious physical markings that would distinguish one region from another, nor are there many instances of genes being expressed in discrete patterns. In fact, a large number of genes that occupy very restricted patterns later in development are first ubiquitously expressed within the disc. As development proceeds, physical landmarks begin to distinguish the major regions of the eye-antennal disc from one another, while gene expression and protein distribution patterns can identify much more discrete domains. Indeed, mosaic clone analysis and transplantation experiments using disc fragments have identified the physical locations of each tissue within the late third larval instar disc [5–9].

Many members of the RD network are expressed throughout the entire disc before being segregated to the eye field. Two poignant examples are the *Drosophila* Pax6 genes, *eyeless* (*ey*) and *twin of eyeless* (*toy*). Starting at stage 15 of embryogenesis and continuing through the first larval instar, both *ey* and *toy* are expressed throughout the entire eye-antennal disc [10,11]. The simultaneous removal of these proteins early in development, when they are universally expressed in the eye-antennal disc, leads to the elimination of the entire disc and all associated head structures that are derived from the disc [12,13]. At the start of the second larval instar, the expression of *ey* and *toy* are restricted to the developing eye field [10,11,14]. If Ey/Toy are necessary for tissue proliferation early in development, then why is expression of both these genes lost from the antennal and head epidermal portions of the disc even as both domains continue to proliferate and grow? The answer may lie in the results from experiments involving the targeted expression of *ey/toy* in the antennal field; in these situations, ectopic eyes are induced [14,15]. It thus appears that Pax6 plays two roles in development: cell proliferation and tissue specification. The changing expression pattern of Pax6 genes over time is critical for separating the two functions of Pax6 in both time and space and ensures that development of the eye-antennal disc and formation of the entire fly head occurs correctly.

To better understand the mechanisms of how the RD network controls tissue specification we analyzed other members that are first expressed throughout the entire eye-antennal disc but are then restricted to just the eye field later in development. Of the many genes that fulfilled this criterion, we focused on *teashirt (tsh)* and its paralog *tiptop* (*tio*) for several reasons. First, the proliferation of the entire eye-antennal disc early in development is partially dependent upon Tsh [12]. Second, both Tsh and Tio induce ectopic eyes in the antenna when over-expressed [16–18]; therefore, their restriction to the eye field later in development is required for proper head formation. And lastly, although *tsh* and *tio* arose through a duplication of an ancestral gene, their temporal/spatial expression patterns are different, and they have distinct protein structures [19–21]. Therefore, we have an opportunity to functionally dissect the roles that these two key factors play within the eye-antennal disc.

Here we show that in the nascent eye-antennal disc Tsh and Toy cooperate to promote cell survival throughout the disc. The simultaneous loss of both factors leads to massive increases in apoptosis, which, in turn, leads to the complete loss of the eye-antennal discs. The adults are headless and die during the pharate pupal stage. The role for Tsh in early disc growth has remained hidden up to this point and is only revealed when Toy protein levels are simultaneously reduced. After the initial growth phase, *tsh* expression is restricted to the eye field. This sequestration is necessary since prolonged expression within the antennal field induces ectopic eye and leg formation [16]. We demonstrate here that during the induction of ectopic eyes Tsh not only initiates expression of retinal selector genes such as *ey*, *sine oculis* (*so*), *eyes absent* (*eya*), and *dachshund (dac)* but it also independently represses the expression of antennal/head epidermal selector genes such as *cut (ct)*, *Lim1*, *aristaless (al)*, and *spineless (ss)*. This supports a model in which the RD network promotes the specification of the eye, in part, by “playing defense” through the suppression of alternate non-ocular fate choices. Forced expression of Tio within the antennal disc leads to similar changes in selector gene expression and tissue fate specification. However, unlike *tsh*, *tio* is never expressed within the antennal field but is always restricted to the undifferentiated cells of the developing eye. Thus, our data indicate that while Tsh/Tio proteins are functionally redundant later in the eye [17,18], sub and neo-functionalization at promoter/enhancer elements are likely to have played a key role in how these genes differentially affect early eye-antennal disc development.

Our findings shed new light on the role that the RD network plays in the development of the eye-antennal field. The RD network first promotes the growth of the entire precursor field before being rewired to promote specification of the retina. This shift in function necessitates a change in expression so that retinal development is confined to the eye field. As many other precursor fields in both flies and vertebrates are parceled out to produce multiple distinct organs, it is likely that similar mechanisms are in place to ensure the fidelity of growth, specification, and patterning.

## Results

### Tsh, but not Tio, alongside Toy is essential for eye-antennal disc development

**Fig 1:**
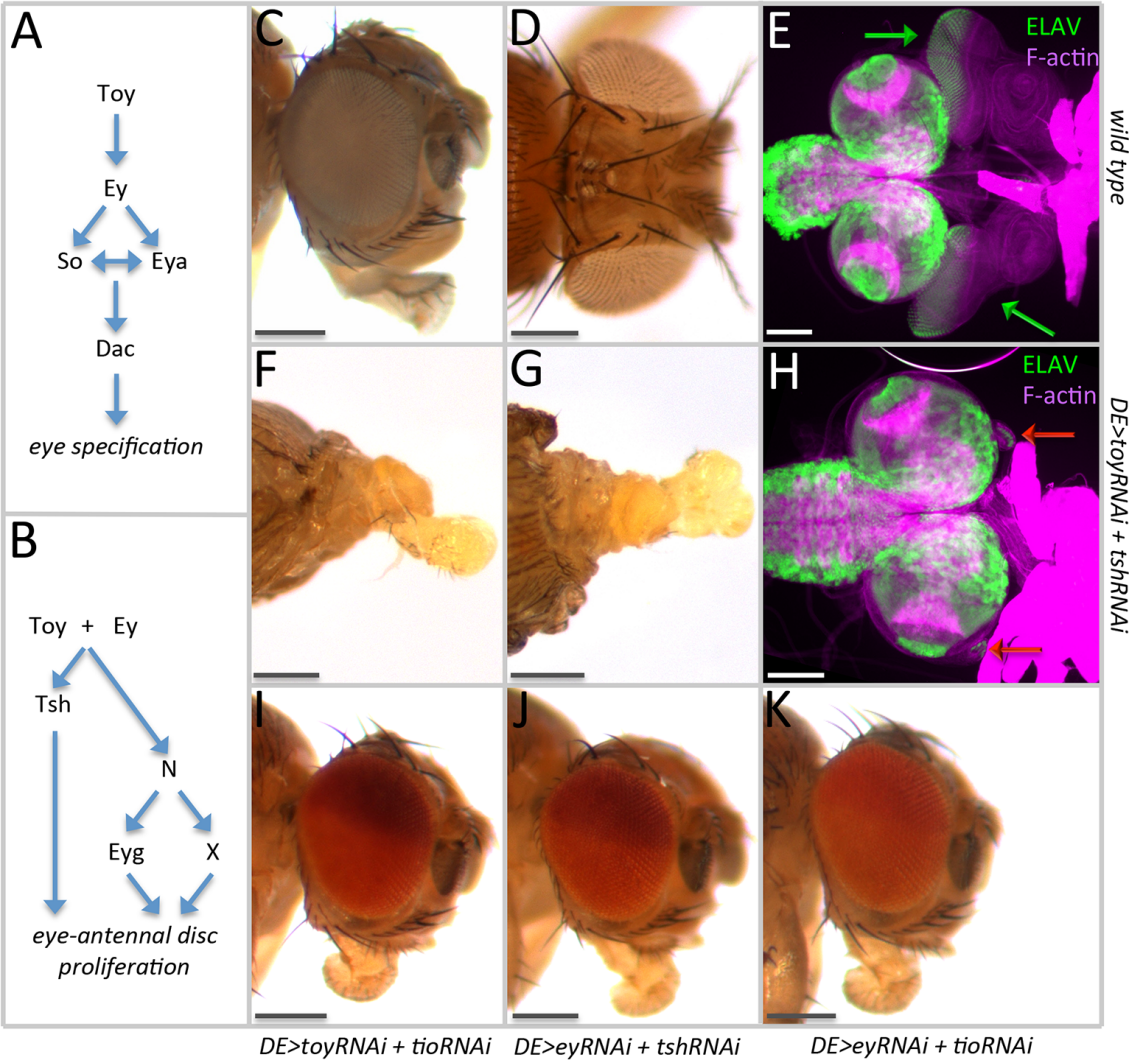
Simultaneous removal of Toy/Tsh eliminates the eye-antennal disc. (A) Schematic diagram of how the RD network regulates eye specification. (B) Schematic diagram of how the RD network might control cell proliferation. (C) Side view of a wild type head. (D) Dorsal view of a wild type head. (E) Light microscope image of a pair of eye-antennal discs (green arrows) from a wild type third instar larva. (F) Side view of a *DE>toy RNAi + tsh RNAi* pharate lethal headless adult. (G) Dorsal view of a *DE>toy RNAi + tsh RNAi* pharate lethal headless adult. (H) Light microscope image of the brain complex from a *DE>toy RNAi + tsh RNAi* third instar larva. The eye-antennal discs (red arrows) are rudimentary in size. (I) Side view of a *DE>toy RNAi + tio RNAi* adult head. (J) Side view of a *DE>ey RNAi + tsh RNAi* adult head. (K) Side view of a *DE>ey RNAi + tio RNAi* adult head. (Scale bars, **100** μm).

The RD network controls both tissue fate specification and cell proliferation in the *Drosophila* eye (Fig 1A and 1B). Tsh, an important member of that network, is expressed throughout the entire nascent eye-antennal imaginal disc before being segregated to the eye field later in development. In the developing eye, Tsh has been implicated in driving both fate specification and cell proliferation [12,22–24]. These conclusions have relied mainly on the use of forced expression assays and protein-protein interaction studies because, due to several technical hurdles, the examination of eye development in *tsh* null mutants has proven to be quite difficult. As a result, there is little understanding of the molecular and developmental roles that Tsh plays during tissue specification and growth. Also, virtually nothing is known about the role that Tsh plays in the nascent disc, prior to the point of parceling of the eye-antennal disc into different individual domains.

To gain insight into the role that Tsh plays in the eye-antennal disc we used a set of RNAi lines to knockdown *tsh* expression during different stages of development. For this study, we used the Dorsal Eye (DE)-GAL4 line, which is an insertion in the (*mirr*) locus [25]. Early in development, DE-GAL4 drives expression in all cells of the eye-antennal disc, but later in development, its expression is restricted to the dorsal half of the eye and a subset of peripodial cells (S1A and S1B Fig) [12]. The use of this GAL4 line allows us to remove *tsh* from the entire eye-antennal disc during embryogenesis and the first larval instar during the period that *tsh* is normally expressed throughout the entire disc. Also, it allows us to, later in development, remove *tsh* from just the eye and compare Tsh negative (dorsal) and Tsh positive (ventral) tissue to each other within the same disc. We extended our study to include Tio since it is a paralog of Tsh and it is functionally redundant to Tsh in several developmental contexts [17,18,20,21]. We also targeted Ey and Toy since Tsh mediates a portion of their growth promoting activity [12].

We first set out to demonstrate the efficacy of the RNAi lines that we are using to target each candidate gene. When DE-GAL4 drives individual RNAi lines during the third larval instar, all four proteins are eliminated from the dorsal half of the eye disc (S1C–S1J Fig) [12]. We also examined transcript levels in late third larval instar eye-antennal discs and, as expected, we see a reduction in expression levels of approximately 50% for *toy*, *tsh* and *tio* (S1K Fig). Unexpectedly, *ey* transcript levels appear unchanged (S1K Fig). We assume that there must be compensation of gene expression within the ventral half of the eye disc through a mechanism that we do not yet understand. Tsh and Tio are thought to mutually repress one another’s expression [17]; therefore, we also looked at expression levels of *tsh* when *tio* is knocked down, and vice versa. There is a 10% increase in *tsh* expression when *tio* is knocked down, and a 32% increase in *tio* expression when *tsh* is knocked down (S1K Fig). Together, these controls demonstrate that the RNAi lines are efficient in eliminating expression of the target genes and that the transcription of *tsh* and *tio* is regulated as predicted by genetic studies.

Our initial results indicate that reducing *ey*, *toy*, *tsh*, or *tio* individually within the DE-GAL4 expression patterns has minimal, if any, effect on eye development (S1C, S1E, S1G and S1I Fig). Consistent, with this finding, transcript levels of the remaining RD genes are not reduced significantly (results are shown just for *so* S1K Fig). While it may seem surprising that the individual loss of these RD genes does not have a phenotype when removed with DE-GAL4, similar findings have been reported previously, and it has been demonstrated that the combined loss of multiple RD genes with this driver will reveal phenotypes that were absent with the single gene knockdowns [12]. Since genetic and biochemical interactions between the *ey/toy* and *tsh/tio* gene pairs exist, we decided to express all possible pairwise combinations of RNAi lines (*toy/tsh*, *ey/tsh*, *toy/tio*, *ey/tio*).

The concurrent reduction of Toy and Tsh eliminates the eye-antennal discs and results in headless adults that die during the pharate stage of pupal development (Fig 1C–1H). This is the very first evidence linking Tsh to early eye-antennal disc development. The headless phenotype is identical to the combined loss of Ey and Toy, and confirms our earlier suggestion that these Pax6 genes and Tsh cooperate to regulate growth of the eye-antennal disc gene [12]. Since our earlier report showed that *tsh* expression is lost when Toy and Ey are eliminated, we suggest that Tsh is functioning downstream of Toy and Ey. Surprisingly, the loss of the eye-antennal discs is specific to the Toy/Tsh knockdown combination, as the Ey/Tsh, Ey/Tio, and Toy/Tio dual knockdowns do not appear to affect eye-antennal disc development (Fig 1I–1K). Loss-of-function *toy* mutants exist and these show defects in the eye-antennal disc [12,26]; therefore, we are confident that the *toy* RNAi lines are behaving as expected. In contrast, eye development in *tsh* null mutants has not been characterized therefore we needed to express the *tsh* RNAi line in another tissue and confirm that the knockdown phenotype is similar to that of the *tsh* loss-of-function mutants. To accomplish this task, we expressed the *tsh* RNAi line within developing wing discs, and reassuringly the adult wings display an outstretched posture that is similar to the *aeroplane-like* allele of *tsh* (S4A Fig) [27,28]. Since the *toy* and *tsh* RNAi lines knock down the expression levels of the target genes and induce the expected loss-of-function phenotypes, our findings indicate that Toy and Tsh cooperate to control the development of the entire eye-antennal disc.

### Toy/Tsh control eye-antennal disc development during embryogenesis and the first larval instar

A previous study has proposed that Tsh is part of a tertiary biochemical complex that includes Ey and Homothorax (Hth) and controls cell proliferation of the developing eye field after segregation of the entire disc [22]. Here we are showing that Tsh and Toy may, together, control additional aspects of eye-antennal disc development. Our model is that Toy and Tsh are functioning prior to the segregation and rise of the different tissues within the eye-antennal disc. Since removal of Ey and Tsh together early in development doesn’t appear to have an effect on the eye-antennal disc, we propose that the Ey-Tsh-Hth complex may be working at a later stage of eye development. To make this determination, we introduced a temperature sensitive GAL80 construct [29] into the *DE-GAL4>UAS-tsh RNAi*, *UAS-toy RNAi* fly strain. GAL80 is an inhibitor of GAL4 activity, and the temperature sensitive nature of the GAL80^ts^ construct allows us to use temperature to modulate the activity of GAL4 temporally and in turn regulate the timing of *toy*/*tsh* RNAi expression. At 18°C (the permissive temperature), GAL80 inhibits the activity of GAL4, thereby silencing the RNAi lines and allowing for normal expression of *toy* and *tsh*. Animals kept at this temperature throughout development emerge as adults that are indistinguishable from wild type. In contrast, GAL80^ts^ is non-functional at 30°C (the restrictive temperature), which allows for the expression of the RNAi lines and the suppression of both genes. At this temperature, larvae lack eye-antennal discs, and the pharate adults are completely headless. By toggling between the permissive and restrictive temperatures, we can temporally control *toy* and *tsh* expression and determine when both genes are required together for disc development. When going back and forth between different temperatures, we had to take into account the dynamics of endogenous protein loss and recovery. We have determined that once animals have been shifted to 30°C and RNAi expression is initiated, it takes roughly 8hrs for Toy [12] and 12hrs for Tsh proteins to be erased from the disc (S2A–S2D Fig). Once flies are returned to 18°C, and the expression of RNAi lines has ceased, it takes approximately 40hrs for Toy [12] and 38hrs for Tsh (S3A–S3F Fig) to return to wild type levels.

**Fig 2:**
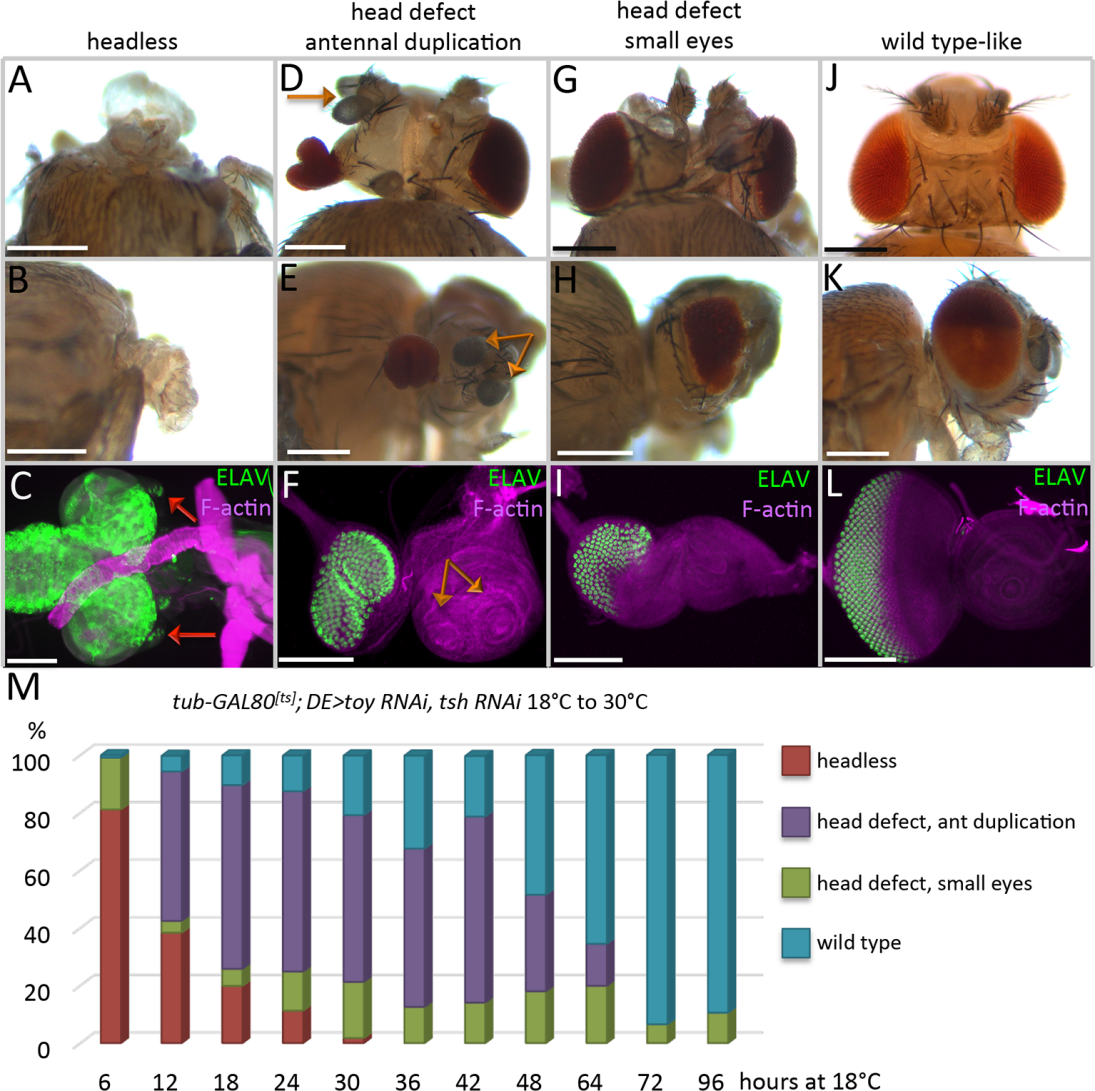
Toy/Tsh are required during successive temporal waves for eye-antennal disc development. (A-M) *tub-GAL80*^*ts*^*; DE>toy RNAi + tsh RNAi*. (A,D,G,J) Dorsal view of adult heads. (B,E,H,K) Side view of adult heads. (C,F,I,L) Light microscope images of eye-antennal discs from third instar larvae. Anterior is to the right. Eye-antennal discs were stained with an antibody that recognizes the pan-neuronal marker ELAV. Removal of Toy and Tsh at different times in development results in four major categories of phenotypes: (A-C) Headless, the red arrows mark the greatly reduced eye-antennal discs. (D-F) Head defects with antennal duplications, the orange arrows mark the duplicated antennal segments. (G-I) Head defects with small eyes. (J-L) Wild type-looking heads and eye-antennal discs. (M) Time-course of Toy/Tsh removal during development. Embryos/larvae were first kept at the permissive temperature of GAL80 (18°C) for the times listed on the X-axis before being shifted to the non-permissive temperature (30°C). Animals were either dissected as late third instar larvae or allowed to reach the pharate adult stage. The graph shows the percentage of animals that have each of the four phenotypes (Y-axis) during each experiment. (N ≥ 100 in each experiment) (Scale bars, **100** μm).

**Fig 3:**
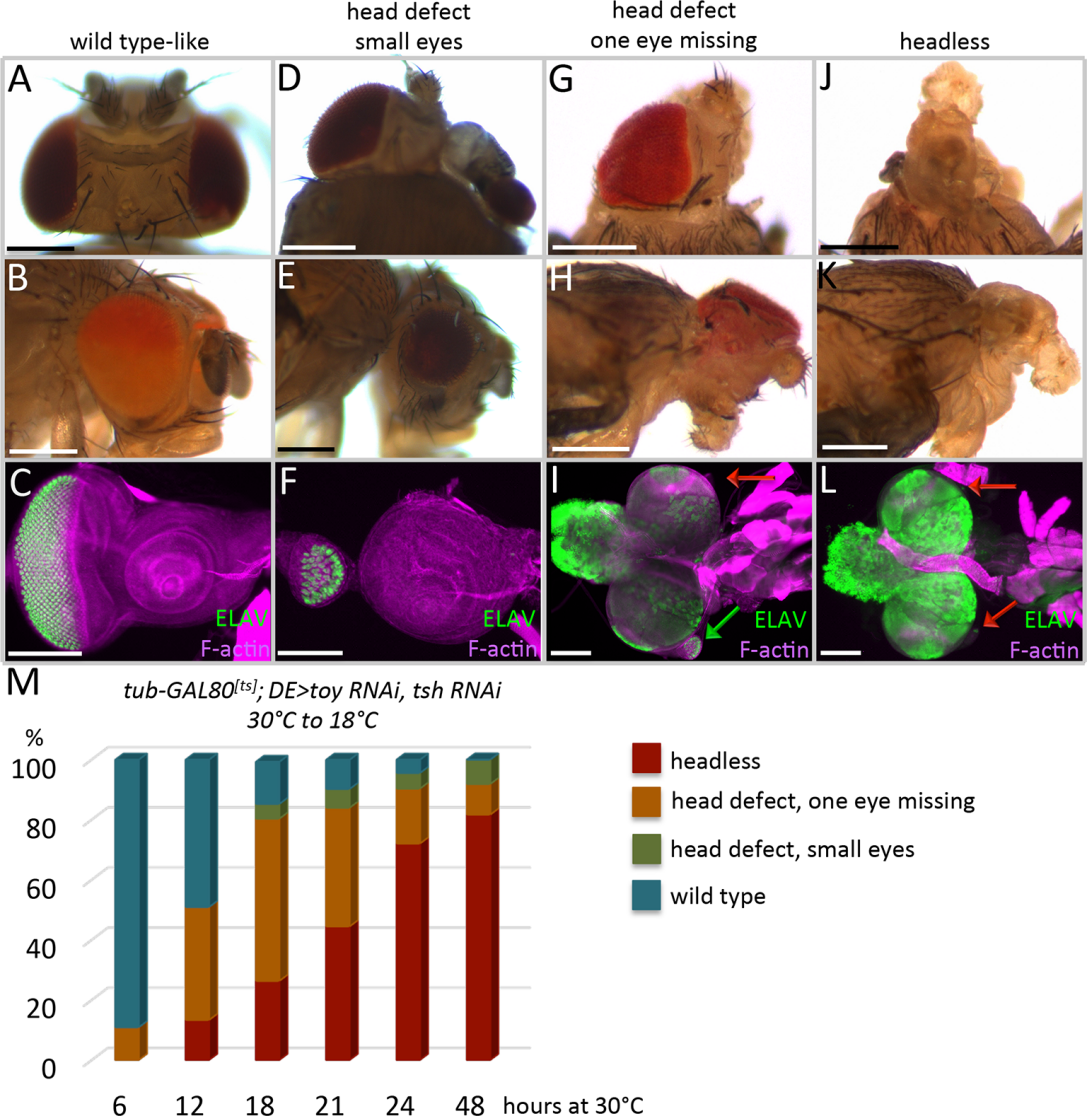
Toy/Tsh are required for growth of the entire eye-antennal eye disc during early larval instars. (A-M) *tub-GAL80*^*ts*^*; DE>toy RNAi + tsh RNAi*. (A,D,G,J) Dorsal view of adult heads. (B,E,H,K) Side view of adult heads. (C,F,I,L) Light microscope images of eye-antennal discs from third instar larvae. Anterior is to the right. Eye-antennal discs were stained with an antibody that recognizes the pan-neuronal marker ELAV. The green arrow marks the presence of partially formed eye disc and the red arrows mark the greatly reduced eye-antennal discs. (M) Time-course for the restoration of Toy/Tsh to the eye-antennal disc. Embryos/larvae were first kept at the non-permissive temperature of GAL80 (30°C) for the times listed on the X-axis before being shifted to the permissive temperature (18°C). Animals were either dissected as late third instar larvae or allowed to reach the pharate adult stage. The graph shows the percentage of animals that were scored (Y-axis) during each experiment. Depending upon the temporal window at which Toy/Tsh are restored to the eye antennal discs, it is possible to either fully (A-C,M) or partially (D-I) rescue defects to the eye, antenna, and head epidermis. However, it becomes exceedingly difficult to restore the eye-antennal disc after the first larval instar. (N ≥ 100 in each experiment) (Scale bars, **100** μm).

We first kept animals at the permissive temperature (18°C) for varying periods of time before shifting to the restrictive temperature (30°C). Depending on when Toy/Tsh are removed, we see a range of phenotypes with headless flies on one extreme and wild type looking flies on the other (Fig 2A–2L). The percentage of flies with more severe defects increases in experiments where the removal of Toy/Tsh begins earlier in development (Fig 2M). We conducted the opposite experiment, in which animals were first kept at the restrictive temperature (30°C) for varying lengths of time before returning them to the permissive temperature (18°C). Again, depending upon the time at which Toy/Tsh proteins are restored, we see a range of mild to severe phenotypes (Fig 3A–3L). The complete loss of the eye-antennal discs is most often associated with longer knockdown periods of *toy/tsh* levels (Fig 3M). After taking into consideration the effect that different temperatures have on developmental rates [30], as well as the time that it takes for Toy/Tsh proteins to either be depleted or recovered, our findings suggest that the critical window for Toy/Tsh control of eye-antennal disc development is between stage 16 of embryogenesis and the end of the first instar (Figs 2M and 3M).

**Fig 4:**
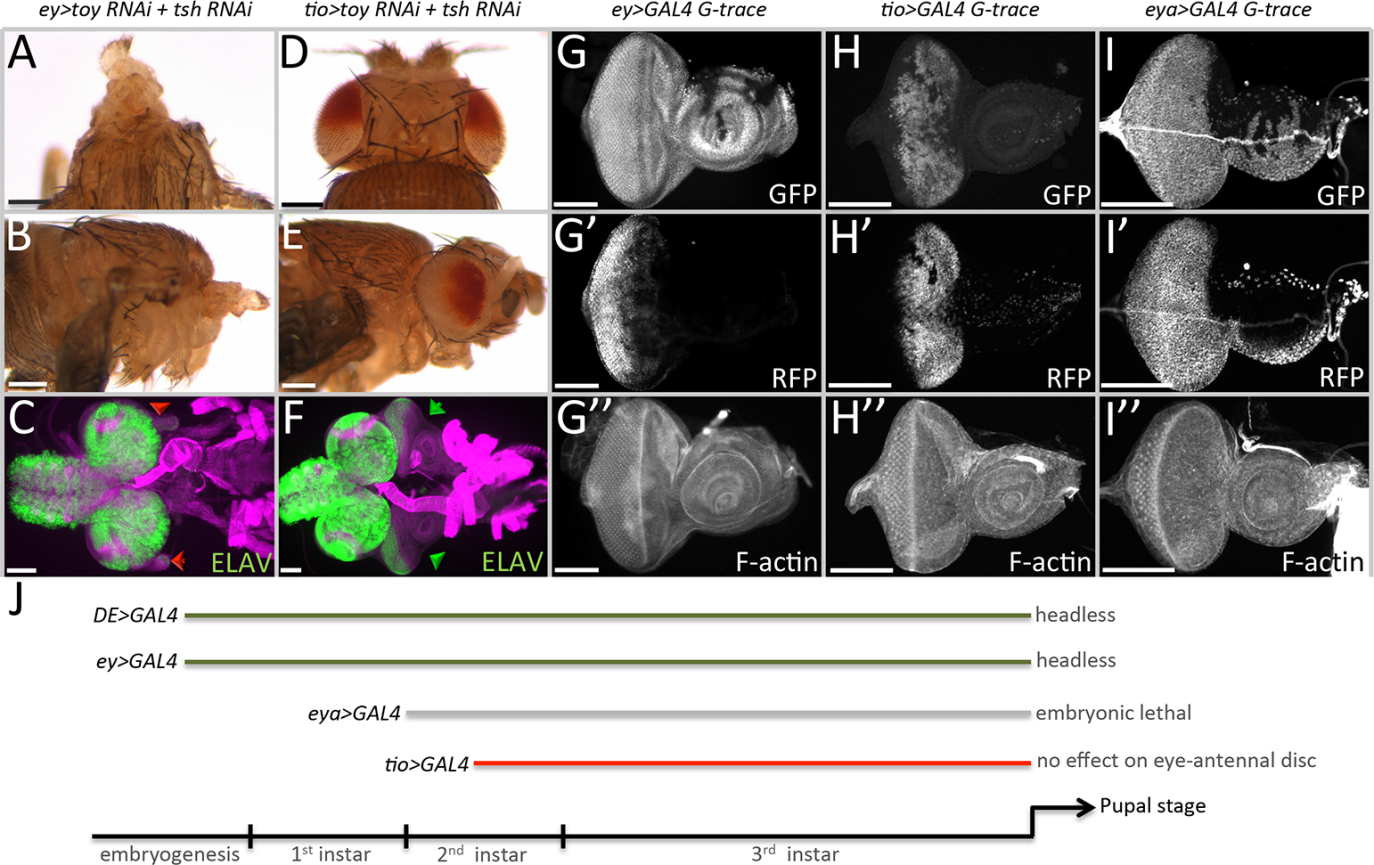
Toy/Tsh are required for growth of the eye-antennal disc prior to the segregation of the eye and antennal fields. (A-C) *ey-GAL4*, *UAS-toy RNAi + UAS-tsh RNAi*. Removal of Toy/Tsh using the *ey-GAL4* driver results in the loss of the eye-antennal imaginal discs and in headless pharate lethal flies. (D-F) *tio-GAL4*, *UAS-toy RNAi + UAS-tsh RNAi*. Loss of Toy/Tsh using the *tio-GAL4* driver has no effect on the development of the eye-antennal disc. (G-I) Light microscope images of third larval instar eye-antennal discs showing historical (GFP) and real-time (RFP) expression patterns of *ey-GAL4* (G), *tio-GAL4* (H) and *eya-GAL4* (I). Anterior is to the right in all images of eye-antennal discs. (J) Schematic depicting the onset of expression of individual GAL4 lines used in this study. Removal of Toy/Tsh using early drivers (*DE-GAL4* and *ey-GAL4*) leads to the loss of the eye-antennal discs and in headless pharate lethal adults. However, removal of Toy/Tsh with the late acting driver (*tio-GAL4*) has no effect on eye-antennal disc development. This suggests that the critical window for Toy/Tsh in promoting growth of the eye-antennal discs lies between stage 12 of embryogenesis and the middle of the second larval instar. These results are consistent with the time-course experiments described in Figs 2 and 3. (Scale bars, **100** μm).

In order to confirm this timeline, we simultaneously knocked down *toy/tsh* using several different GAL4 drivers that initiate their expression at various times in development. Expression of the *toy/tsh* RNAi lines with *ey*-GAL4, which is first activated during embryogenesis, also results in larvae lacking eye-antennal discs and pharate stage pupae that are headless (Fig 4A–4C, 4G and 4J). This effect is synergistic, as expression of the individual RNAi lines with *ey*-GAL4 has much milder effects on the eye and head (S4B–S4E Fig). In contrast, removing Toy/Tsh simultaneously or individually with a *tio*-GAL4 driver, whose expression in the eye begins during the mid-second larval instar, does not affect the eye-antennal disc or the adult head (Fig 4D–4F, 4H and 4J, S4H–S4K Fig). *tio*-GAL4 also drives expression in regions within the leg disc that will give rise to the adult pleura and coxa (S4L Fig). If Tsh is removed from these regions (*tio-GAL4*, *tsh RNAi*), then the leg discs are duplicated (S4M–S4P Fig). The resulting adult legs are considerably smaller than their wild type counterparts and are often internalized within the adult fly (S4N–S4P Fig). This phenotype is consistent with the known role of Tsh in *Drosophila* leg development [31,32]. Based on the leg phenotypes associated with the *tio-GAL4*, *UAS-tsh* RNAi strain, the lack of observable phenotypes in the eye-antennal disc is due to the late onset of the *tio* enhancer.

We also tried to remove Toy/Tsh with an *eya*-GAL4 driver, which begins its expression at the start of the second larval instar (Fig 4I) [33]. While individual knockdown of *toy* with this driver does not affect the disc (S4F and S4G Fig), the knockdown of *tsh* resulted in embryonic lethality, therefore, we cannot draw any conclusions from this experiment. Unfortunately, there are no other suitable drivers to further test the temporal requirements of *tsh/toy*. However, the results from *DE-GAL4*, *ey*-GAL4, and *tio*-GAL4 are consistent with the GAL80^ts^ time course experiments and indicate that Toy/Tsh function together during late embryogenesis and through the first larval instar to promote growth of the eye-antennal disc. This timeline for Toy/Tsh is consistent with the critical period for Ey/Toy function and is consistent with data that places *tsh* genetically downstream of both Pax6 genes [12]. Our findings are also consistent with a role for Tsh in promoting growth as proposed by [22], where Tsh is thought to work with Ey and Hth to keep the cells in proliferation. An important difference is that these two studies are addressing how Tsh interacts with distinct Pax6 proteins and at different times in development.

### Increased cell death is the principal cause of the headless phenotype in *toy/tsh* mutants

**Fig 5:**
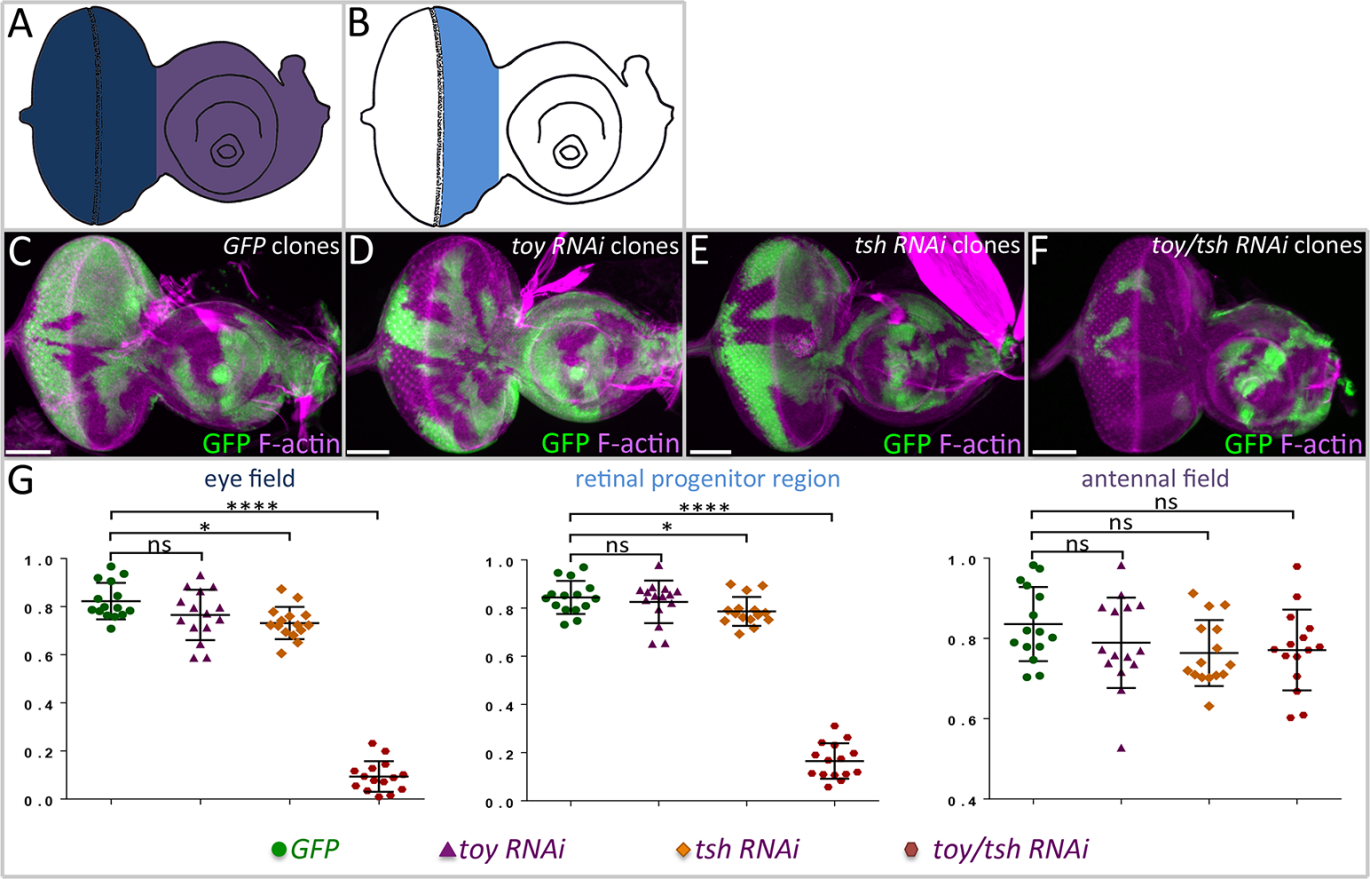
Loss of Toy/Tsh in clones leads to a loss of tissue growth. (A,B) Drawings of third larval instar eye-antennal discs in which the entire eye field (dark blue), retinal progenitor region (light blue) and antennal field (purple) are demarcated. (C-F) Light microscope images of eye-antennal discs containing *wild type* (C), *toy RNAi* (D), *tsh RNAi*, and *toy/tsh RNAi* clones–all clones are marked by GFP. Anterior is to the right. (G) Comparison of clone size in the eye field, retinal progenitor domain, and antennal field. The individual loss of Toy or Tsh has no effect on the size/growth of the clone. However, the combined loss of Toy/Tsh inhibits clone growth within the progenitor region and eye field. As the expression of both genes is withdrawn from the antennal disc by the end of the first larval instar, there is no noticeable effect on clone size within the antenna. N = 15 per genotype, *P ≤ 0.1, **P ≤ 0.01, ***P ≤ 0.001, ****P ≤ 0.0001 (Scale bars, **50** μm).

We then used the flp-out over-expression system to generate mosaic clones in which *toy* and *tsh* RNAi lines were simultaneously expressed in smaller cell populations. This allows us to measure the effect that the loss of both genes has on tissue growth. The size of the *toy*/*tsh* double knockdown clones and clones in which each gene had been knocked down individually were compared to GFP control clones. The growth of each type of clone was examined within the entire eye field (Fig 5A, dark blue), the antennal field (Fig 5A, purple), and just the retinal progenitor region (Fig 5B, light blue). Within the antennal field, control, individual RNAi, and double RNAi-expressing clones are recovered with equal frequency and are of nearly identical sizes (Fig 5C–5G). This is most likely due to the fact that expression of both *toy* and *tsh* become segregated to the eye field during the second larval instar, thus rendering the expression of the RNAi lines largely ineffective later in development. In contrast, within the eye field and retinal progenitor region, clones in which both *toy* and *tsh* were simultaneously knocked down are rare and significantly smaller than either control or individual RNAi-expressing clones (Fig 5C–5G). These findings are consistent with the above experiments using DE-GAL4, where we see that the loss of individual factors has no effect on growth but the concurrent loss of both Toy/Tsh appears to either block cell proliferation, induce apoptosis, or both.

Since several RD genes are known to participate in promoting growth and blocking cell death [12,22,23,33–39], we asked if the loss of Toy/Tsh proteins affects the expression of any of these other network members. This experiment is important for determining if Toy/Tsh are working directly on cell proliferation/survival pathways or if they are working through other RD network members. We analyzed the expression of *ey*, *eya*, *eyg*, *dachshund* (*dac*), and *hth* in clones lacking both Toy and Tsh, and in all cases, the expression patterns and levels of these genes were unaffected (S5A–S5F Fig), suggesting that the regulation of growth by Toy/Tsh is likely independent of the known RD gene network.

We then attempted to determine if the headless phenotype is caused by a lack of cell proliferation, an increase in apoptosis, or both. To do this, we examined the cell cycle profile of Toy/Tsh deficient cells. Since DE-GAL4 drives expression only in the dorsal compartment later in development, we can use EdU staining and PH3 to look at potential differences in S phase and M phase populations within the dorsal (mutant) and ventral (wild type) compartments. Despite detecting significantly fewer total cells in the mutant tissue (Hoechst positive), we do not observe any significant difference in the densities of S and M phase cells between wild type tissue (ventral) and tissues that lack both proteins (dorsal, S6A–S6H Fig), and as such did not pursue any rescue experiments with cell proliferation-promoting genes.

**Fig 6:**
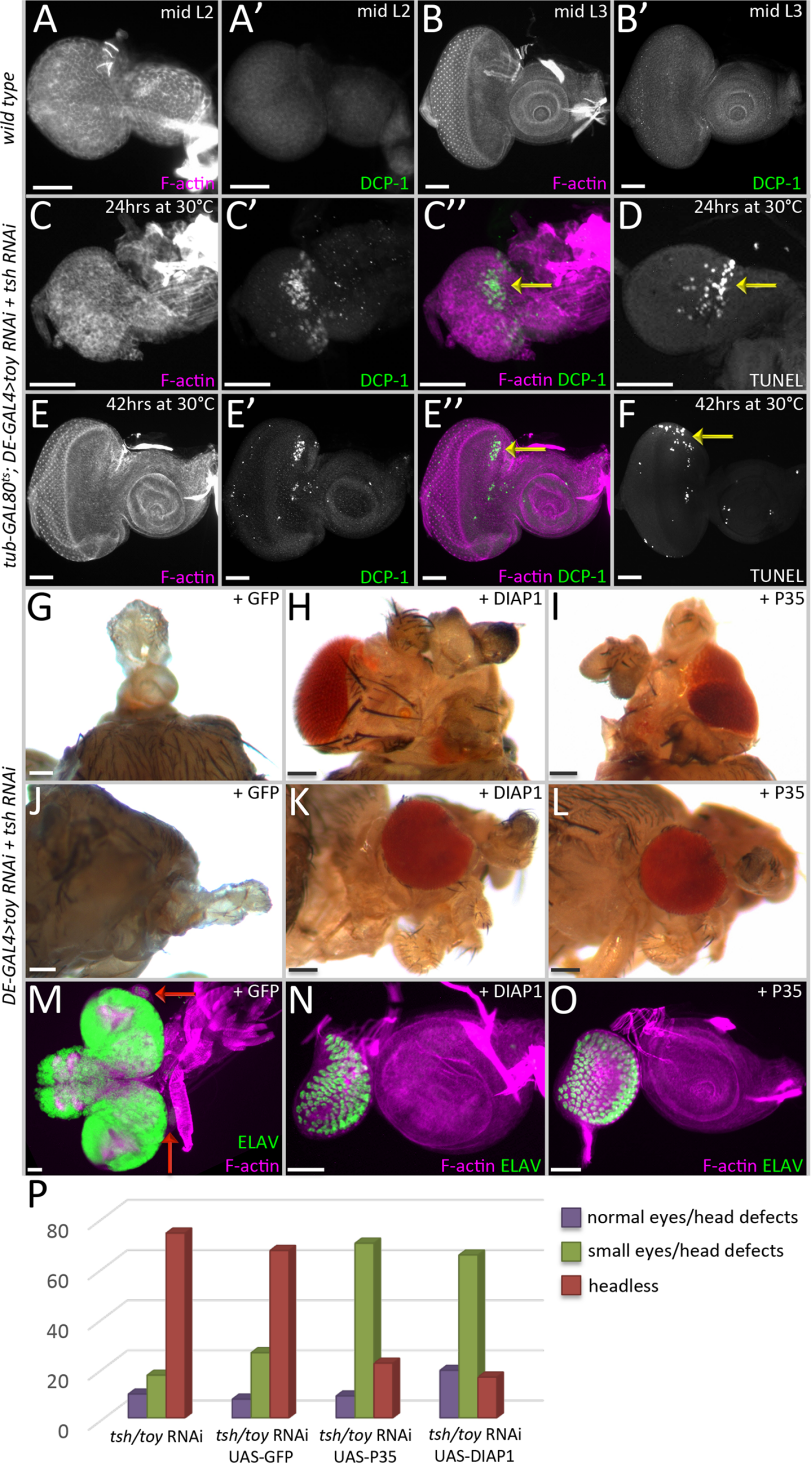
Cell death is a leading cause of the headless phenotype in *tsh/toy* mutant animals. (A-B) light microscope images of wild type second (A) and third (B) larval instar discs. Note that the DCP-1 is almost undetectable in wild type. (C-F) Light microscope images of discs showing apoptotic cells in the dorsal compartment (yellow arrows) where Toy and Tsh are removed. (A-D) *tub-GAL80*^*ts*^*; DE>toy RNAi + tsh RNAi* (C,D) 24hrs at 30°C (E,F) 42hrs at 30°C. (G-L) Dorsal and side views of adult heads in which expression of P35 and DIAP1 has partially restored eye and head development. (M-O) Light microscope images of eye-antennal discs showing partial restoration of the eye-antennal disc. (G,J,M) *DE-GAL4*, *UAS-toy RNAi*, *UAS-tsh RNAi*, *UAS-GFP*. (H,K,N) *DE-GAL4*, *UAS-toy RNAi*, *UAS-tsh RNAi*, *UAS-DIAP1*. (I,L,O) *DE-GAL4*, *UAS-toy RNAi*, *UAS-tsh RNAi*, *UAS-P35*. (P) Graph quantifying the degree of rescue when cell death is blocked. (N ≥ 25 in each experiment) (Scale bars, **50** μm).

Using TUNEL staining and antibodies against the cell death marker Dcp-1 [40], we observed dying cells in the dorsal half of the eye when Toy/Tsh levels are reduced (Fig 6C–6F, yellow arrows), while wild type discs do not show any dying cells (Fig 6A and 6B). In this experiment, the RNAi lines were expressed only later in development to ensure that there is an actual disc to examine. By the time we start inducing RNAi expression, expression of the DE-GAL4 driver has been already segregated to the dorsal half of the eye, so that is why cell death is only observed in the dorsal compartment. We then asked whether we could restore eye and head development to the *toy/tsh* double knockdown animals by blocking cell death via expression of two apoptosis inhibitors, DIAP1 and P35 [41,42]. In both cases, substantial restoration of the eye and head were observed (Fig 6H, 6I, 6K, 6L, 6N, 6O and 6P). This is in contrast to the higher percentage of animals lacking any rescue that is seen when a control UAS-GFP transgene is expressed in the double knockdown background (Fig 6G, 6J, 6M and 6P). In total, this set of results suggests that Toy/Tsh are suppressing apoptosis during the early stages of eye-antennal disc development and that the reduced size of double mutant clones and headless phenotypes are mainly the results of the inappropriate induction of cell death. Our findings are consistent with the ability of the mammalian TSHZ3 protein to suppress the expression of the cell death caspase gene, *CASP4* [43].

### Expression of *tsh/tio* transforms the arista into eyes, tarsal legs, and head epidermis

**Fig 7:**
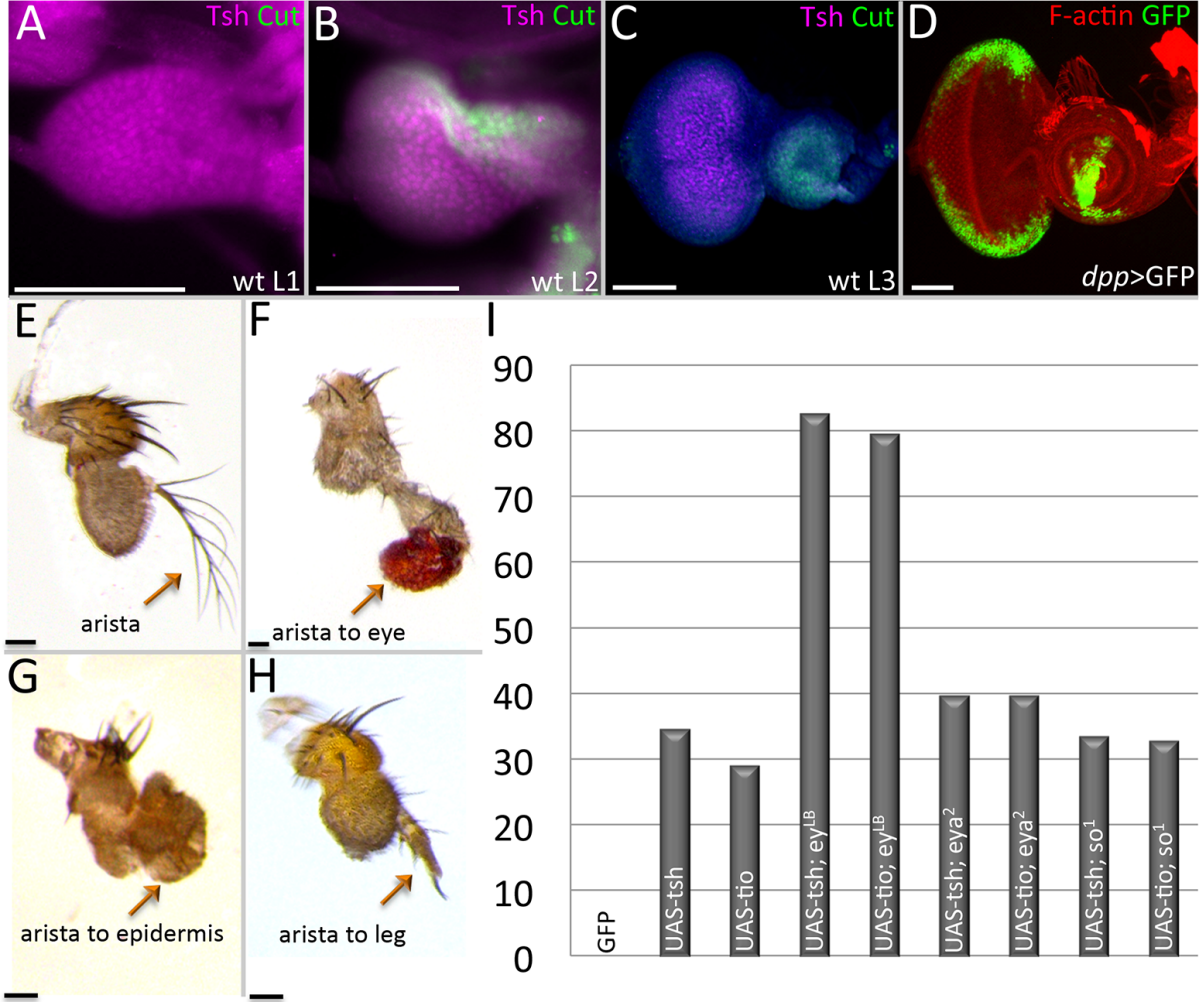
Continued expression of *tsh/tio* in the antennal disc alters its fate. (A-D) Light microscope images of eye-antennal discs. (A) Wild type first instar eye-antennal disc. Tsh protein is expressed throughout the entire disc. Cut protein is not yet detected. (B) Wild type second instar eye-antennal disc. Tsh protein continues to be found throughout the disc while Cut protein is found within the antennal portion. (C) Wild type third instar eye-antennal disc. Tsh protein is found just within the undifferentiated cells of the eye field while Cut protein is exclusively seen in the antennal field. (D) *dpp-GAL4*, *UAS-GFP*. The *dpp-GAL4* construct drives expression along the posterior-lateral margins of the eye field and within a ventral sector of the antennal disc. (E-H) Light microscope images of adult antennal segments. (E) Wild type antenna with arista. (F) *dpp-GAL4*, *UAS-tsh*; the arista has been converted into an ectopic eye. (G*) eya*,^*2*^ *dpp-GAL4*, *UAS-tsh*; the arista is transformed into head epidermal mass. (H) *dpp-GAL4*, *UAS-tsh*, *ey*^*LB*^; the arista has been transformed into the leg tarsal segment. (I) Chart documenting the percentage of arista to leg transformations when *tsh/tio* are expressed in wild type and RD gene mutants (N ≥ 35).(Scale bars, **50** μm).

From the results we have shown above, we know that Tsh and Toy are required to promote growth of the eye-antennal disc by suppressing apoptosis. Then why are these proteins segregated to the eye field and removed from the antennal and head epidermis fields later in development? The answer appears to lie in the shifting roles that these proteins play in development. During the second larval instar, both Toy and Tsh proteins are restricted to the retinal progenitor zone ahead of the advancing morphogenetic furrow (Fig 7A–7C) [14,22,24]. If either protein is allowed to remain in the antennal field, then ectopic eyes are induced [14,16]. Having said that, we propose that there is a critical difference between how Ey/Toy and Tsh/Tio group of proteins bring about this tissue transformation. Ey/Toy are transcriptional activators, while Tsh/Tio are transcriptional repressors, and this inherent difference dictates how they act upon tissue-specific genes and ultimately, bring about alterations in tissue fate. Current models propose that induction of *tsh/tio* causes activation of *ey* and this further activates the expression of several downstream RD genes such as *eya*, *so*, and *dac*. This cascading set of events ultimately induces the formation of an ectopic eye [16,18]. An example is presented in S7A Fig, in which the forced expression of *tsh* induces both *eya* expression and ectopic eye formation.

To separate the role(s) of Tsh/Tio from other RD members in inducing ectopic eye formation, we used a dpp-GAL4 (Fig 7D) driver to induce *tsh/tio* expression within the antennal and head epidermal fields of wild type as well as *ey*^*LB*^, *eya*^*2*^, and *so*^*1*^ loss-of-function mutants. Each mutant is characterized by the absence or severe disruption of the eye that is caused by deletions of eye-specific enhancers [35,44] (S11 Fig). The loss of these enhancers completely eliminates expression of each gene within the developing eye. It also prevents ectopic eye formation from being induced by the expression of *tsh/tio* [16]. We observe that targeted expression of *tsh/tio* can induce the transformation of the arista into ectopic eyes, tarsal leg segments, or a mass of head epidermal tissue [16,18] (Fig 7E–7H). The ability to transform a portion of the antenna into the homologous leg segment is consistent with a report showing that Tio functions as a selector gene for promoting leg development in the milkweed bug, *Oncopeltus fasciatus* [45].

Surprisingly, blocking eye formation through mutations in *ey*, *so*, and *eya* does not revert the transformed tissue back to an arista as one might have expected. Instead, the percentages of the other types of homeotic transformations rise compensating for the loss of the arista-eye fate switch. For example, if *ey* is removed (while *tsh/tio* is still expressed), then arista–leg transformations increase to 80% from a starting point of 30% (Fig 7I). Similarly, if *so* or *eya* are removed, while expression of *ey* and *tsh*/*tio* is maintained (S9A–S9F Fig), then the number of arista–head epidermis transformations rises, while the arista–leg fate drops back to about 35% (Fig 7I).

### Tsh/Tio in the antenna down-regulates non-ocular antennal and head epidermal genes

**Fig 8:**
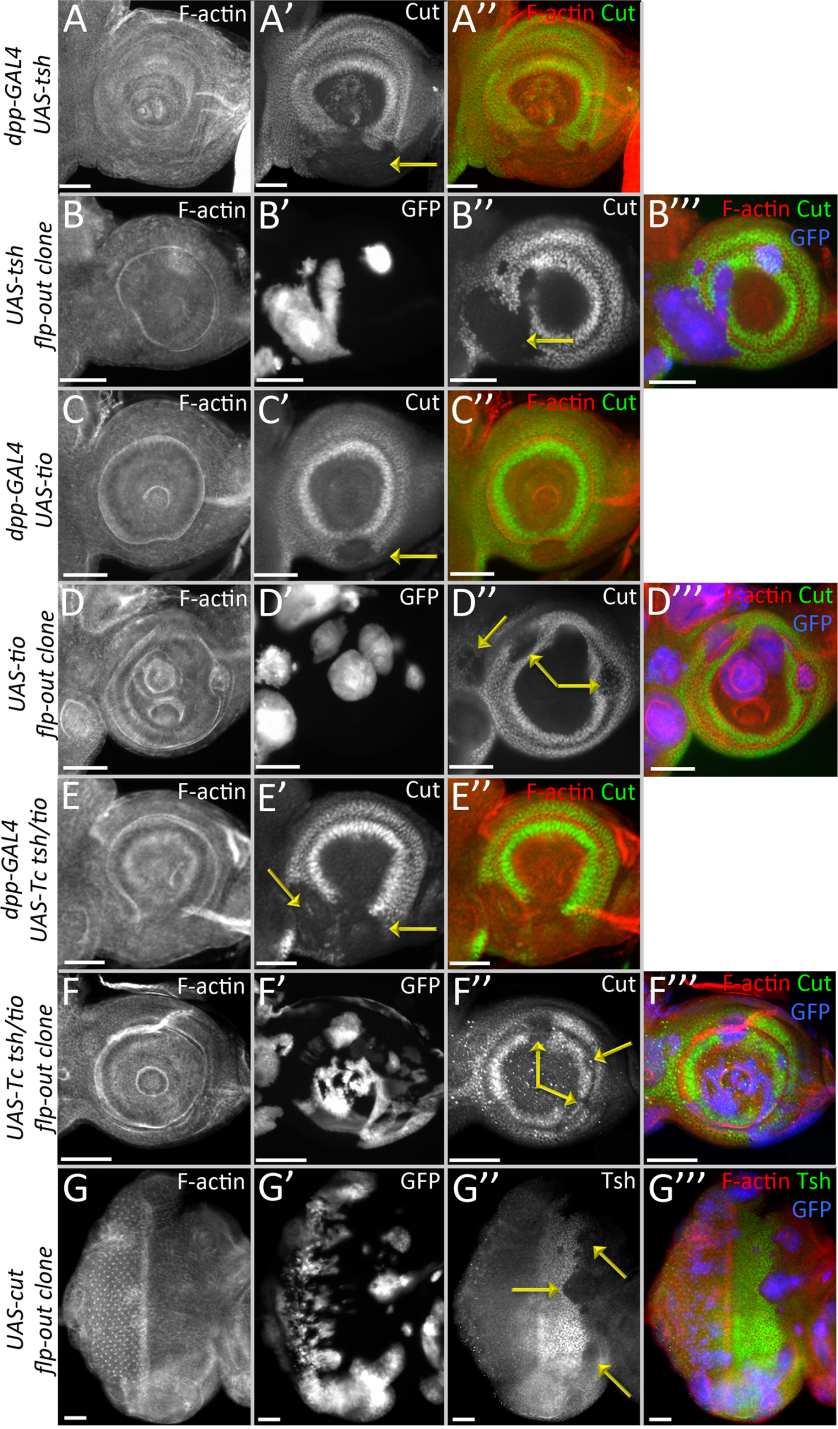
Continued presence of Tsh/Tio in the antennal disc inhibits *cut* expression. (A-G) Light microscope images of antennal discs. (A,C,E) Expression of Drosophila *tsh* (A), *tio* (C), or Tribolium *tsh/tio* (E) using *dpp-GAL4*. In all cases *cut* expression is inhibited within the *dpp-GAL4* domain. (B,D,F) Expression of Drosophila *tsh (B)*, *tio (D)*, or *Tribolium tsh/tio* (F) using flp-out over-expression clones. (G) Expression of Drosophila *cut* using flp-out over-expression clones. *tsh* expression is inhibited in the clones. Yellow arrows in each panel demarcate areas where *cut*(A-F) or *tsh*(G) expression is repressed. Posterior is to the right. (Scale bars, **50** μm).

With the knowledge that Tsh/Tio are transcriptional repressors, we sought out to seek answers to two main questions—how does continued expression of *tsh/tio* in the antennal field affect the antennal/head epidermis genes, and why is it that we observe these distinct phenotypes on induction of *tsh/tio* when different RD network members are removed. We used *cut*, (*ct*), a gene necessary for sensory organ formation in the antenna, as the readout for our primary assay [46]. Expression of either *tsh* or *tio* in the antennal field inhibits the expression of *cut* (Fig 8A–8D, yellow arrows) [47–49]. This finding is consistent with a study showing that Tsh/Tio negatively regulates *ct* expression during renal tubule development [50], suggesting that the observed cross-regulatory relationship is not tissue-specific and is in fact maintained throughout the multiple tissues. Moreover, the ability to repress *ct* is at least 250 million years old, as the *tsh*/*tio* homolog in the red flour beetle, *Tribolium castaneum*, can also inhibit *ct* expression when it is forcibly expressed within the *Drosophila* antennal disc (Fig 8E and 8F, yellow arrows). We also observe that the opposite is true–induction of *ct* expression inhibits expression of *tsh* in the eye progenitor region (Fig 8G, yellow arrows). This is also consistent with the regulatory relationship between Tsh-Cut in the renal tubules [50].

Tsh and Tio are zinc (Zn) finger transcription factors [21,51], and in a prior study, we deleted each Zn finger individually and showed the differential use of these DNA/protein binding domains in the context of inducing ectopic eyes and promoting cell proliferation [20]. Here, we expressed those deletion constructs within the antenna in an attempt to determine which Zn finger might be necessary for inhibiting *ct* expression. None of the individual deletion proteins were compromised in their ability to repress *ct* expression (S8A–S8G Fig, orange arrows), which suggests that the Zn finger domains may function redundantly in this context to repress antennal/head epidermis genes.

### Tsh/Tio requires the PLDLS domain in order to repress head epidermal genes

**Fig 9:**
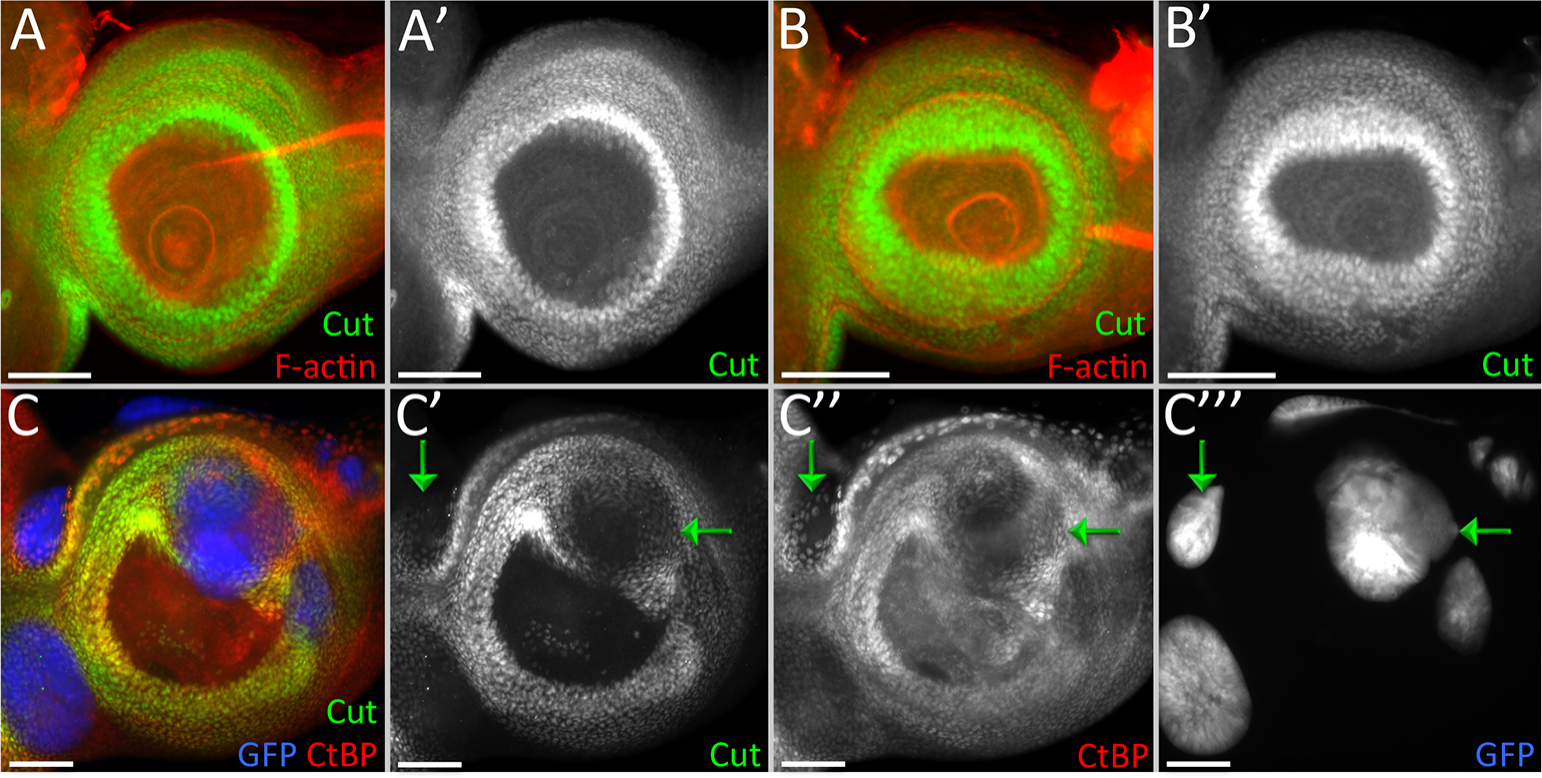
CtBP may not be the only co-repressor assisting in Tsh/Tio mediated repression. (A-C) Light microscope images of antennal discs. (A,B) Expression of Drosophila TshΔPLDLS (A) or TioΔPLDLS (B) using *dpp-GAL4*. In both cases, loss of PLDLS domain compromises the ability of Tsh and Tio to repress *cut*. (C) On expression of *tsh* in *CtBP* mutant MARCM clones, where the clones are labeled in blue (GFP), *cut* is still repressed (C’). Posterior is to the right. (Scale bars, **50** μm).

Apart from the zinc finger domains, Tsh/Tio each have an additional conserved protein motif, the PLDLS domain. The co-repressor protein, C-terminal Binding Protein (CtBP), interacts with the PLDLS domain to mediate the repressive activity of proteins that contain PLDLS and some limited variants. To test if Tsh/Tio induced repression of *ct* requires the PLDLS domain, we expressed modified Tsh/Tio proteins in which the PLDLS domain has been removed (Tsh ΔPLDLS/Tio ΔPLDLS). The removal of the PLDLS domain eliminates the ability of both Tsh and Tio to repress *cut* (Fig 9A and 9B). We then tested if CtBP is the actual repressor that mediates the inhibitory effect of Tsh/Tio. To do this, we used the MARCM method to express a full-length Tsh protein within *CtBP* null mutant cells. We found that Tsh is still capable of repressing *ct* (albeit at reduced levels) in the absence of CtBP (Fig 9C, green arrows). This suggests that there may be additional factors within the *Drosophila* genome that interact with the PLDLS domain and aid in the repressive activity of Tsh/Tio proteins.

### Tsh/Tio repression of *cut* expression is independent of the Ey/So/Eya module

**Fig 10:**
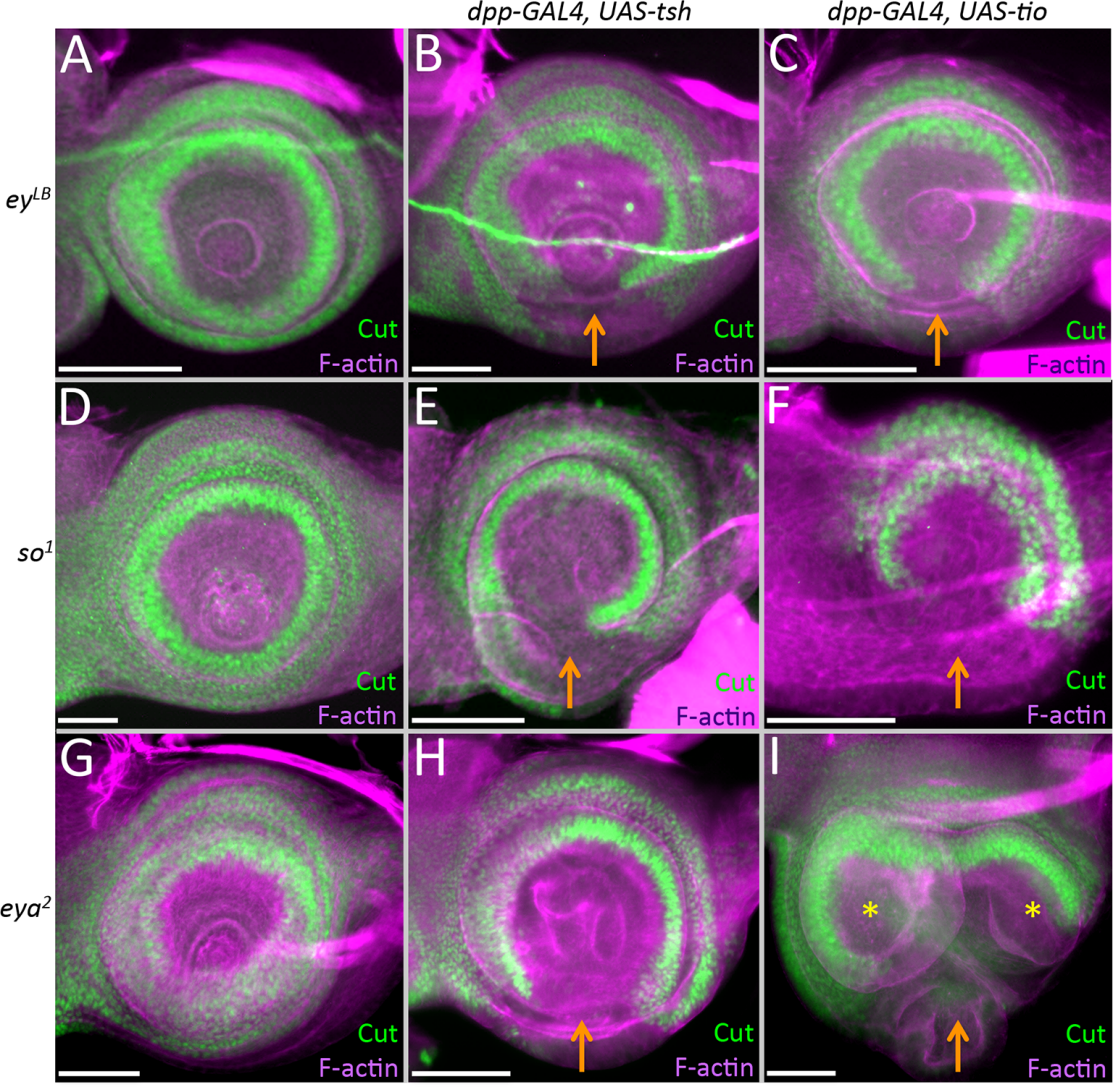
Repression of *cut* by Tsh/Tio is independent of the retinal determination network. (A,D,G) *cut* expression in the antennal disc of *ey*^*LB*^, *so*^*1*^, and *eya*^*2*^ mutant antennal discs. Note that in these mutants *cut* expression is identical to wild type. (B,C,E,F,H,I) Loss of *cut* expression when *tsh* (B,E,H) or *tio* (C,F,I) continues to be expressed in the antenna with *dpp-GAL4*. Orange arrows demarcate zones where *cut* expression is down regulated. Yellow asterisks mark the duplicated antennal segments. Posterior is to the right. (Scale bars, **50** μm).

Other RD member genes have been implicated in the inhibition of antennal/head epidermis genes such as *ct* [13,52–54], so we set out to determine if the repression of *ct* is due to Tsh/Tio directly or if it is the indirect consequence of activating one or more of these other retinal network genes instead. As is the case in wild type discs, *ct* is expressed normally within the antenna of *ey*^*LB*^, *eya*^*2*^, and *so*^*1*^ mutants (Fig 10A, 10D and 10G). However, it can be repressed in these mutant backgrounds when either *tsh* or *tio* is forcibly expressed (Fig 10B, 10C, 10E, 10F, 10H and 10I). This suggests that Tsh/Tio represses *ct* independently of the core *ey/so/eya* module. Further evidence to support this conclusion comes from three observations. First, although Ey has been implicated in the repression of *ct*, it can only do so when an ectopic eye is induced (S7B and S7C Fig). It should be noted that Tsh and Tio are activated in the ectopic eyes that are induced by Ey. Second, Ey is unable to repress *cut* in the absence of *so* (S7D Fig). And lastly, forced expression of either *eya* or *so*, individually, is insufficient to inhibit *ct* expression (S7E and S7F Fig). There is some debate surrounding the last point as very high levels of So can repress *ct* expression [13]. Overall, these data suggest that other RD genes may not be directly responsible for silencing *ct* in the eye disc. Instead, Tsh/Tio might be the primary agents responsible for preventing non-ocular selector genes from being expressed within the eye field.

### Down-regulation of *ss* by Tsh/Tio is correlated with the arista–leg transformation

**Fig 11:**
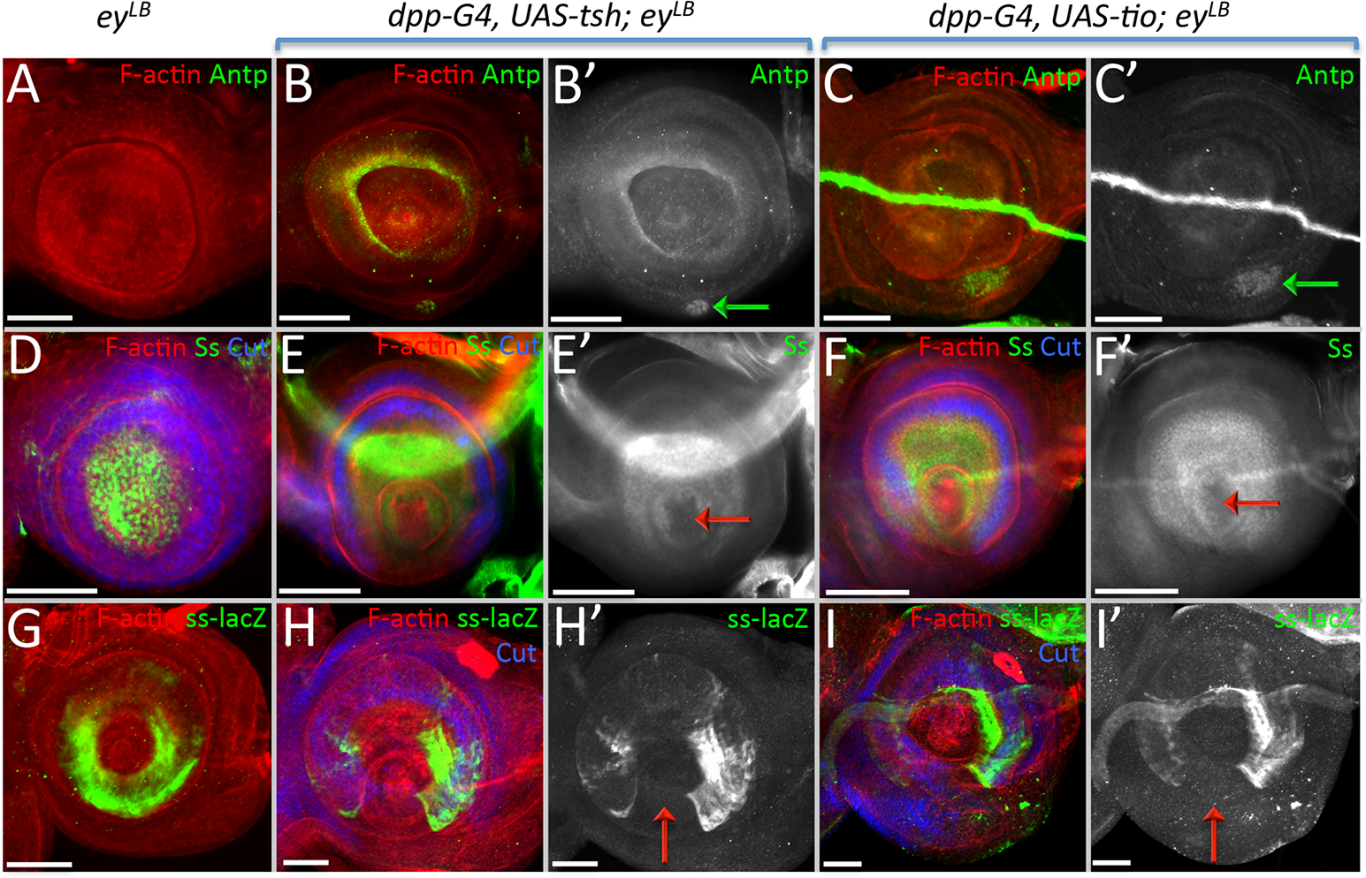
(A-I) Light microscope images of third larval instar antennal imaginal disc. Posterior is to the right in all antennal disc images. (A,D,G) *ey*^*LB*^ (B,E,H) *dpp-GAL4*, *UAS-tsh; ey*^*LB*^ (C,F,I) *dpp-GAL4*, *UAS-tio; ey*^*LB*^ (A) *Antp* is not expressed normally in the antennal field. (B,C) Tsh/Tio are capable of activating *Antp* expression (green arrows). (D) Ss protein is present throughout the entire aristal segment. (E,F) Tsh/Tio repress *ss* expression within a portion of the aristal segment (red arrows). (G) A *ss-lacZ* construct is expressed within a subdomain of the aristal segment. (H,I) Tsh/Tio are capable of repressing the activity of the *ss* antennal enhancer (red arrows). (Scale bars, **50** μm).

As mentioned above, unlike other RD members, the induction of *tsh/tio* exhibits a unique set of phenotypes, including an arista-leg transformation. This was particularly interesting to us, so we set out to identify the molecular mechanism underlying this transformation. The Wingless (Wg) pathway is important for specifying head epidermal and antennal fates [55–58] and it is activated strongly within the aristal segment (S9G Fig), so we first asked if Tsh/Tio are silencing the Wg pathway. In disagreement with this model, the expression of Tsh/Tio actually induces Wg signaling (S9H Fig, blue arrow). A second potential mechanism might involve the Hox gene *Antennapedia* (*Antp*). Mis-expression of *Antp* within the antenna causes a complete antenna-leg transformation [59], we then asked if *Antp* is activated when *tsh/tio* is misexpressed. *Antp* is not normally expressed in either wild type or *ey*^*LB*^ mutant eye-antennal discs [60] (Fig 11A). If *tsh/tio* are forcibly expressed in the antennae of wild type discs, *Antp* expression remains silenced (S9I and S9J Fig). However, *Antp* expression is activated within the antennal field when *tsh*/*tio* are expressed in *ey*^*LB*^ mutants (Fig 11B and 11C green arrows). However, *Antp* is activated within the ventral head epidermis (Fig 11B and 11C green arrows), which is not the position from which the arista is formed. Thus, in this case, we don’t believe that Antp is mediating the arista–leg transformation. We also examined the possibility that the loss of *ct* is responsible for the arista-leg transformation, but expression of a *ct* RNAi construct with *dpp-GAL4* did not induce a change in tissue fate (S9K–S9M Fig).

We actually believe that the arista–leg transformation that we are seeing is caused by a loss of *spineless* (*ss*) expression. We pursued this model since the Tsh/Tio induced arista–leg transformation that we observe is reminiscent of transformations that characterize the *aristapedia* allele of *ss* (*ss*^*a*^) [61,62]. Ss protein is distributed within the aristal segment in wild type discs (Fig 11D). However, if *tsh*/*tio* are expressed within the *dpp*-GAL4 domain, then *ss* expression is lost in the arista (Fig 11E and 11F, red arrows). A transcriptional reporter (*ss-lacZ*) that drives expression within the aristal segment [63] is also responsive to Tsh/Tio (Fig 11G–11I), suggesting that both genes are acting on the *ss* aristal enhancer. While it is not clear if either Tsh or Tio is directly binding to the enhancer, our model is that the arista–leg transformation is due to an inhibition of *ss* expression by Tsh/Tio. Additionally, we have evidence suggesting that the repression of *ss* is independent of *Distalless (Dll)* and *hth*, two key antennal selector genes that are upstream regulators of *ss* [62,64–66]. The expression of neither gene is affected when *tsh*/*tio* are expressed within the antenna (S9N–S9S Fig) thus Tsh/Tio are likely to repress *ss* directly.

We also considered the regulatory relationship between Tsh/Tio and *Lim1* and *aristaless (al)*, both of which are expressed in the distal-most segments of the antenna and leg [67,68]. Upon induction of Tsh/Tio, *al* and *Lim1* are always lost within the ventral head epidermis in both wild type and *ey*^*LB*^ mutant antennal discs (S9T–S9Y Fig, S10A–S10C Fig). However, expression of these two factors is sometimes maintained within the aristal segment. In looking at the aristal segments more carefully we noticed that if Ey is not activated in the arista, then both *Lim1* and *al* expression are maintained in this segment (S10A and S10B Fig). However, if Ey is activated in the arista, then both *al* and *Lim1* are lost. We predict that the adult heads derived from these discs will harbor the arista–eye transformation (S10C, Fig 7F). A more complicated situation involves Dac, which is lost in the A3 ring (S9Z–S9BB Fig, orange arrows) but activated in the ventral head epidermis (S9Z–S9BB Fig, green arrows). Our results suggest that transformation of ventral head epidermis into eyes and the arista into eyes, legs, and head epidermis is the result of two distinct but equally important events: (1) activation of the RD network and (2) inhibition of an endogenous non-retinal gene regulatory network. The type of transformation is dependent upon a combinatorial code of transcription factor activation and silencing.

## Discussion

Throughout the animal kingdom developing tissues often give rise to multiple distinct organs. Examples that we have mentioned earlier in this paper include the vertebrate anterior forebrain, as well as the wing and eye-antennal discs of *Drosophila*. Other examples include the mammalian diverticulum, which gives rise to the lungs, trachea, and larynx, and the *Drosophila* leg discs, which not only produce the legs themselves but also portions of the thorax. Our understanding of how initially uniform cellular fields generate multiple organs has been dominated by models in which different GRNs (which include batteries of transcription factors and multiple signaling pathways) become activated in discrete domains at key stages of development. Each GRN is then tasked with specifying the size and the primary fate of each organ, preventing each tissue from adopting the wrong fate, and with ensuring that all requisite cell types are specified and positioned correctly. Such models are challenged by several questions and observations. First, in a variation of the proverbial chicken versus egg conundrum–one must consider if the localized GRN is initially specifying the domain, or has the domain been already specified by upstream factors and the localized GRN is simply carrying out a pre-determined set of instructions. Second, it has been observed that in many instances members of the localized GRN are actually expressed throughout the entire tissue. Therefore, it is not clear how tissue fates are allocated if GRNs are uniformly expressed and competing with each other within the same populations of cells to activate and repress different fates?

**Fig 12:**
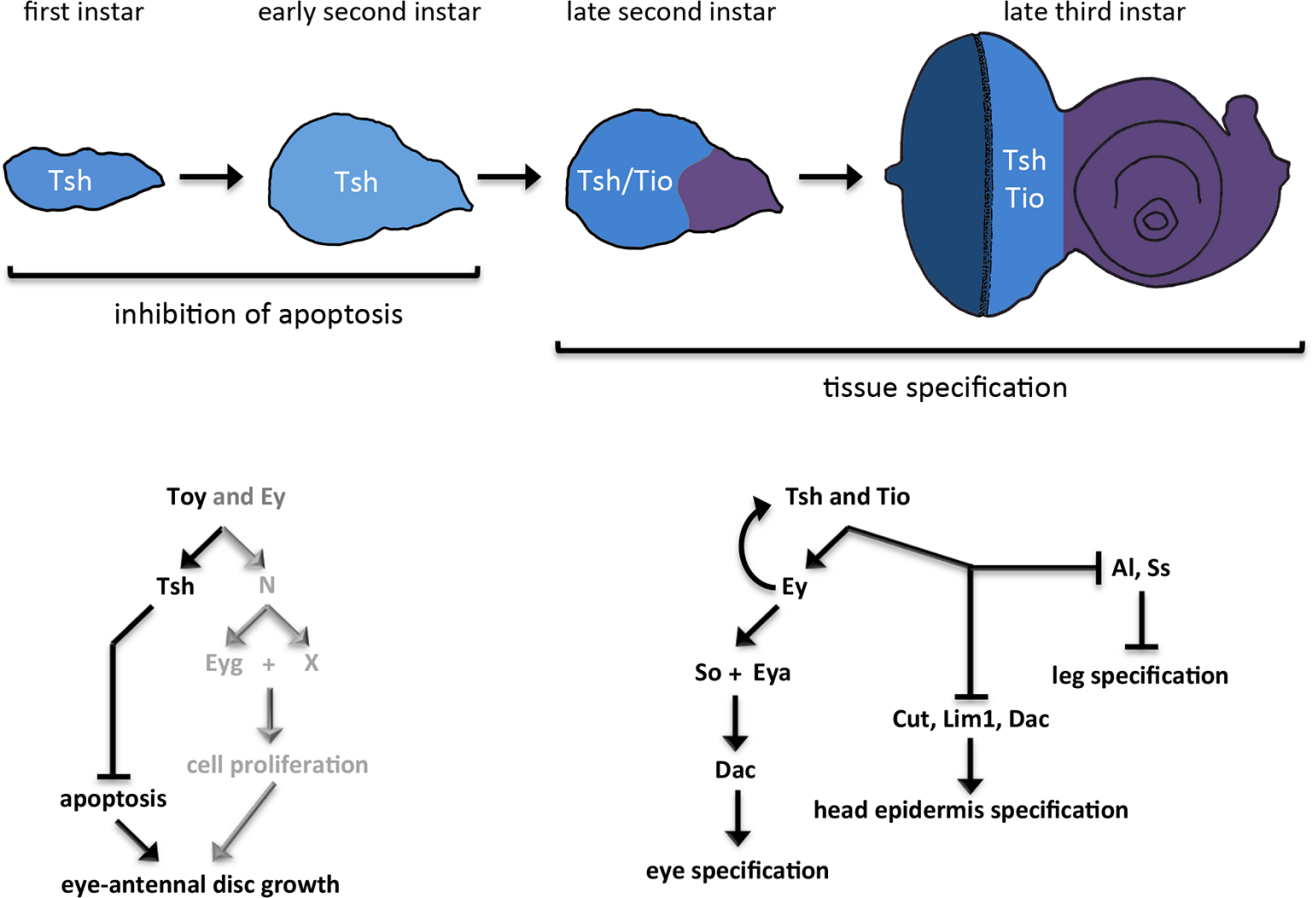
Summary of the role that Tsh/Tio play in the eye-antennal disc. Within the first and early second larval instars, *tsh* is expressed throughout the entire eye-antennal disc. During these developmental stages, Tsh cooperates with Toy to suppress apoptosis. In their combined absence, the entire eye-antennal disc is lost. Prior findings from our group have indicated that N/Eyg function in parallel to Tsh to promote cell proliferation throughout the entire disc (grey lettering) [12]. The Pax6 proteins Toy and Ey function upstream of all three Tsh, N, and Eyg (this report) [12]. During the late larval second instar, *tsh* expression is restricted to the eye disc and *tio* expression is initiated (also within the eye disc). By the third larval instar both proteins are restricted to the undifferentiated cells ahead of the advancing morphogenetic furrow. If Tsh/Tio remains in the antennal field during these later developmental stages they induce ectopic eyes, suppress head epidermis development, and relieve the default repression on leg development.

In this paper, we provide one mechanistic solution to the latter paradigm. Here, we provide a genetic and developmental mechanism for how a uniform field can be parceled into distinct territories by gene regulatory networks that are initially expressed throughout the entire field. In our example, we have used the *Drosophila* eye-antennal disc to show that the retinal determination GRN is rewired temporally and spatially during development. In the nascent disc, several members of the RD network promote the growth of the entire field by promoting cell proliferation and cell survival (Fig 12, left panel). As development proceeds, the expression of these genes is sequestered within the developing eye field where they continue to promote tissue growth. In addition, these genes are now tasked with promoting the fate of the eye while simultaneously blocking the field from adopting non-ocular fates (Fig 12, right panel).

This last feature of the RD network, namely the repression of non-ocular fates, is a relatively new idea and is supported by recent findings in the *Drosophila* eye-antennal disc and the vertebrate anterior forebrain. In flies, mutations within the RD network result in a homeotic transformation of the eye into either an antenna or head epidermis [10,13,33,52–54]. These two non-ocular fates are also derived from the eye-antennal disc. Similarly, disruption of the equivalent RD network results in a transformation of the eye into the hypothalamus, diencephalon, and telencephalon [69–74]. These tissues are, along with the eye, derived from adjacent territories within the anterior forebrain. Together, these findings suggest that the RD network “plays defense” during development and prevents the eye from adopting the fates of adjacent tissues. It is likely that all GRNs play similar roles in their respective tissues. For example, *hth* is a critical member of the head epidermal GRN in *Drosophila* and *hth* loss-of-function mutations result in a head epidermis to eye transformation [75,76]. Given that the five different GRNs that operate in the eye-antennal disc (one each for the eye, antenna, ocellus, head epidermis, and maxillary palp) are likely promoting primary fates and repressing all other fates, our findings underscore the need for all GRNs members to be segregated to their respective domains prior to the tissue specification period.

**Fig 13:**
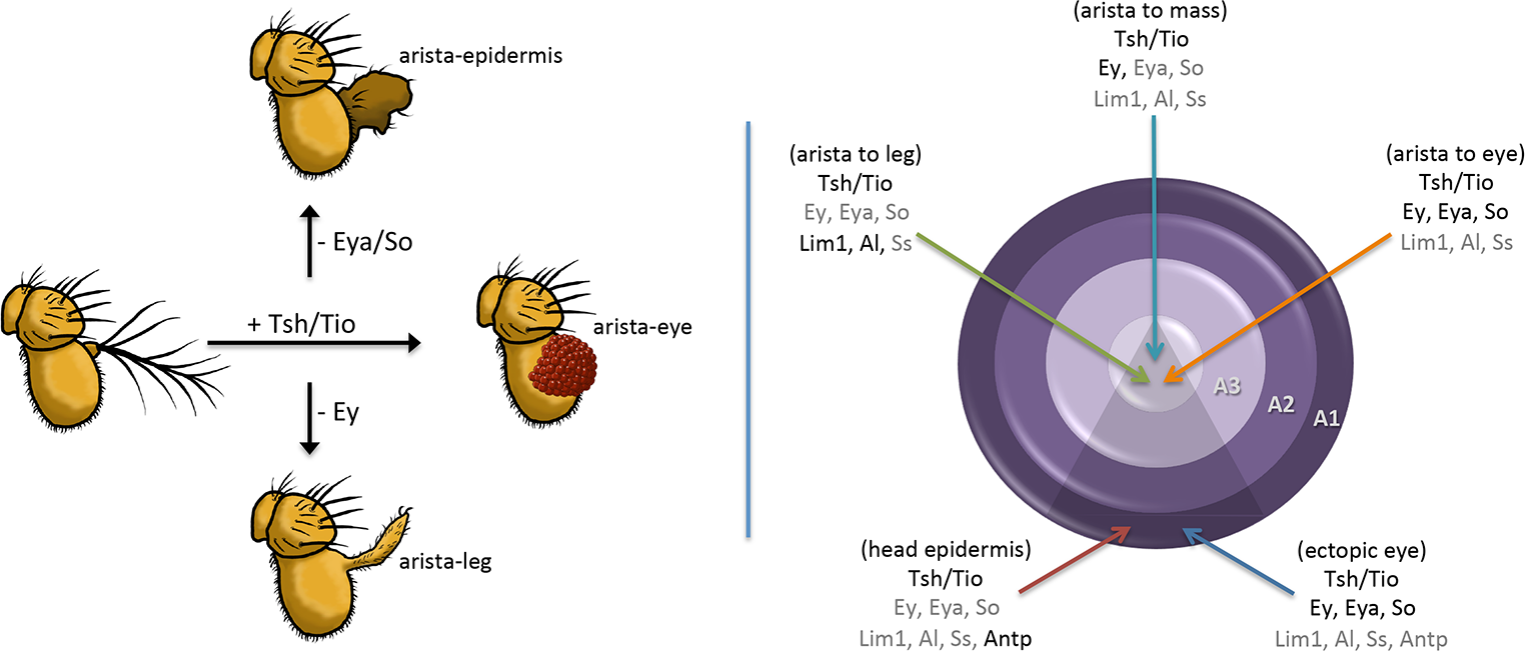
Summary of Tsh/Tio dependent antennal transformations. (Left panel) If Tsh/Tio remain in the antennal disc, then the arista can be transformed into an ectopic eye. Similar transformations of the ventral head epidermis are also observed (not depicted). If *ey* expression is eliminated, then Tsh/Tio transform the arista into the tarsal portion of the leg. Interestingly, if *ey* expression is maintained but transcription of downstream members such as *eya* and *so* are lowered, then the arista does not take on a particular alternate fate but instead grows as head epidermal mass. (Right panel) The positions of the various transformations that are caused by the presence of Tsh/Tio in the antennal discs are depicted. Included are the on/off states of a number of eye, antennal, and head epidermal genes. Genes coded in black are in the “on” state while genes listed in grey are in the “off” state. Our results indicate that the arista–leg transformation appears to be due to the loss of *ss* rather than an up-regulation of *Antp*. The repression of antennal and head epidermal genes by Tsh/Tio appears to occur independently of the Ey/Eya/So module of the RD network.

In the example that we have provided here, the continued expression of Tsh/Tio proteins with the antenna and head epidermis (beyond the point at which they are normally restricted to the eye field) results in several distinct homeotic transformations: head epidermis to eye, arista to eye, arista to tarsal leg and arista to head epidermis (Fig 13 left). The wide range of transformation types is due to the diversity in selector gene activation and repression by Tsh/Tio (Fig 13, right). These results illustrate the drastic and deleterious consequences of inappropriate GRN member expression.

Our study demonstrates that nature has evolved an elegant mechanism for utilizing selector genes for different developmental tasks. In our example, members of the RD GRN are used early to promote the growth of multiple adjacent tissues while later the same GRN is used to promote the fate of one tissue while repressing all other tissue fates. This is gracefully achieved by allowing some RD GRN members to be expressed throughout the entire eye-antennal disc early in development while sequestering the entire network to the developing eye field during the process of tissue specification. Evidence from other GRNs within *Drosophila* and vertebrates suggest that this temporal and spatial rewiring may be a universally used genetic and developmental mechanism.

## Materials and methods

### Fly stocks

The following fly stocks were used in this study: (1) *DE-GAL4* (Georg Halder), (2) *ey-GAL4* (BDSC), (3) *tio-GAL4* (Kwang Choi), (4) *eya-GAL4*, (5) *dpp-GAL4* (Graeme Mardon), (6) *hsFLP*^*22*^ (BDSC), (7) *Actin5C>GAL4* (BDSC), (8) *Actin5C>y*^*+*^*>GAL4*, *UAS-GFP S65T* (BDSC), (9) *Actin5C>y*^*+*^*>GAL4*, *UAS-lacZ* (BDSC), (10) *UAS-tsh* RNAi (BDSC), (11) *UAS-tio* RNAi (BDSC), (12) *UAS-ey* RNAi (BDSC), (13) *UAS-toy* RNAi (BDSC), (14) *tub-GAL80*^*ts*^ (BDSC), (15) UAS-RedStinger, UAS-FLP, Ubi-p63E(FRT.STOP)Stinger (BDSC), (16) *UAS-GFP* (BDSC), (17) *UAS-ey*, (18) *UAS-eya*, (19) *UAS-so*, (20) *UAS-tsh*, (21) *UAS-tio*, (22) *UAS-tsh ΔZnF1*, (23) *UAS-tsh ΔZnF2*, (24) *UAS-tsh ΔZnF3*, (25) *UAS-tio ΔZnF1*, (26) *UAS-tio ΔZnF2*, (27) *UAS-tio ΔZnF3*, (28) *UAS-tio ΔZnF4*, (29) *UAS-tsh ΔPLDLS* (30) *UAS-tio ΔPLDLS* (31) *UAS-Tc tsh/tio*, (32) *UAS-tsh* (Amit Singh), (33) *UAS-eyg* (BDSC), (34) *UAS-p35* (BDSC), (35) UAS-*DIAP1* (BDSC), (36) *eyg-GFP* (BDSC), (37) *eya*^*2*^ (Nancy Bonini), (38) *so*^*1*^ (Larry Zipursky), (39) *ey*^LB^, (40) *ss* 522^*(1–5)*^-*lacZ* (Ian Duncan), (41) FRT82B *tub-GAL80* (BDSC), (42) FRT82B *CtBP*^87De-10^ (Yutaka Nibu), (43) *Wg* signaling GFP reporter (3xGRH-4TH-GFP) (Ken M. Cadigan). BDSC = Bloomington Drosophila Stock Center. Stocks without a donor have been generated in our lab. All crosses (except for GAL80 temperature shift experiments) were conducted at 25°C.

### Antibodies and microscopy

The following primary antibodies and stains were used: chicken anti-β-gal (1:800, Abcam), guinea pig anti-Toy (1:500, Henry Sun), guinea pig anti-Ss (1:100), mouse anti-Ey (1:100, DSHB), mouse anti-Cut (1:100, DSHB), mouse anti-Eya (1:4, DSHB), mouse anti-Dac (1:100, DSHB), mouse anti-Antp (1:100), mouse anti-Dll (1:500, Dianne Duncan), rabbit anti-GFP (1:1000, Invitrogen), rabbit anti-cleaved Dcp-1 (1:100, Cell Signaling Technologies), rabbit anti-Tsh (1:3000, Stephen Cohen), rabbit anti-Hth (1:1000, Richard Mann), rabbit anti-PH3 (1:20,000, Abcam), rabbit anti-Lim1 (1:1000, Juan Botas), rat anti-ELAV (1:100, DSHB), rat anti-Al (1:1000, Gerard Campbell), guinea pig anti-dCtBP (1:500) Yutaka Nibu, Hoechst 33342 (1:2000, Invitrogen). Developmental Studies Hybridoma Bank = DSHB. Secondary fluorophore-conjugated antibodies and phalloidin-fluorophore conjugates (for detection of F-actin) are from Molecular Probes. Imaginal discs were prepared as described previously in [77]. TUNEL Assay (Sigma-Aldrich) was performed as per manufacturer’s instructions. For adult antennae, adult fly heads were removed using a surgical blade and were placed in a concave depression glass slide containing isopropanol. Using forceps, the antennae were then dissected away from the adult heads while the tissue incubated in isopropanol. The dissected antennae were then allowed to air dry and then mounted on a glass slide using Permount (Fisher Scientific). Adult eyes, heads, and antennae were imaged with a Zeiss Discovery Light Microscope or a Leica M205FA Stereo Microscope.

### GAL80 inducible expression system

*tub-Gal80*^*ts*^*; DE-GAL4*, *toy RNAi*, *tsh RNAi* embryos were first collected for an hour at either 18°C or 30°C as per the experiment and then further incubated at the egg laying temperature for defined periods of time before being transferred to the opposite temperature. Eye-antennal imaginal discs were dissected at specific time points after the temperature shift or from third instar larvae.

### Clonal induction and data analysis

The heat shock flp-out over-expression system was used to induce clones overexpressing *tsh* RNAi and/or *toy* RNAi. Embryos of the appropriate genotype were collected after an egg laying period of 1hr at 25°C. Clones were induced at 24hrs after egg lay (AEL) by a single heat shock pulse of 15min in a 37°C water bath. Larvae were returned to 25°C, and eye-antennal discs were dissected at specific time points. For each genotype clones in three different regions were measured: eye region, antennal region, and eye progenitor region (see Fig 5A and 5B). Adobe Photoshop CS 5.1 was used to outline and measure the area of the clones induced in the eye-antennal disc (in pixels). Statistical significance was calculated using one-way ANOVA with GraphPad Prism. *tsh* over-expression/*CtBP* mutant MARCM clones were induced with a 20-min heat pulse in a 37°C water bath 24 hrs AEL. Larvae were returned to 25°C, and eye-antennal discs were dissected from third instar larvae.

### EdU incorporation and PH3 assay

Click-iT EdU Alexa Fluor 555 imaging Kit (Invitrogen) was used to detect the cells in S phase. The protocol provided by the manufacturer was adapted and standardized for the eye-antennal imaginal discs. First, the eye-antennal discs were dissected in PBS and incubated in 50 μM EdU containing PBS for 15mins. This was followed by fixation in Paraformaldehyde-Lysine-Periodate (PLP) fixative as described in [77]. The fixed discs were then sequentially washed with 0.1% TritonX PBS and 3% BSA containing PBS. Next, eye-antennal discs were incubated with Click-iT reaction cocktail as per the manufacturer’s instructions followed by washing in 3% BSA containing PBS and 0.1% TritonX PBS. This was followed by standard immunostaining with pH3 antibody to detect the cells in M phase. Finally, for nuclear staining, the eye-antennal imaginal discs were stained with Hoechst 33342. Eye-antennal discs were imaged using Leica SP5 confocal. Total cell numbers, as well as EdU and PH3 positive cell density, were measured using Imaris Image Analysis Software. Statistical significance was calculated using a paired t-test.

### RT-qPCR

RNA from wild type (control), *DE>toy* RNAi, *DE>ey* RNAi, *DE>tsh* RNAi, and *DE>tio* RNAi eye-antennal imaginal discs was isolated as described by [33] and subjected to RT-qPCR analysis as described in [78]. For each genotype, RNA isolation, cDNA synthesis, and qPCR were performed on three separate biological replicates with each sample consisting of approximately 50 third larval instar eye-antennal imaginal discs.

The following primers were used to detect *toy*, *ey*, *tsh*, *tio* and *so* transcripts using RT-qPCR: *toy* F: 5’-CCA GAG GCA CGT ATT CAG GTT TGG-3’; *toy* R: 5’-TTA TTT GCC GTG CTG GTT CGA C-3’ (QuantPrime) [79]; *ey* F: 5’-TGG TAG GTC AAT CAC CCA ACC-3’; *ey R*: *5’-*GCT GCT GTA GTG CCT GAT GG-3’; *tsh* F: 5’-TCG CAC CAA TCT TTA TGG AAG G; *tsh* R: GTA CCT ACA GAG AGA TCG AGT GG-3’; *tio* F: 5’-GAG GCC GTC ATG CTG GAA AT-3’; *tio* R: 5’-ATG CGA CTC ATT CGA TGG ACA-3`; *so* F: 5’-GCC TGT GTT TGC GAG GTT CT-3-; *so* R: 5’-TGC AGC TTA TCA CAT TGT GGC-3’ (FlyPrimerBank) [80].

## Supporting information

S1 Fig The RNAi lines used in this study are efficacious in reducing target gene transcripts and protein levels. (A,B) Light microscope images showing the lineage tracing (A) and real time (B) expression of the *DE-GAL4* driver. Since GFP (lineage marker) is present in all cells of the imaginal disc (A), the driver should be expressed throughout the entire disc at some point in development. In a previous study [12] we confirmed that *DE-GAL4* driver is expressed throughout the disc during embryogenesis and the first larval instar. (C,E,G,I) Light microscope images of adult heads. (D,F,H,J) Light microscope images of third instar eye discs. (C,D) *DE>toy RNAi* (E,F) *DE>ey RNAi* (G,H) *DE>tsh RNAi* (I,J) *DE>tio RNAi*: expression of each RNAi individually has no effect on the overall structure of the adult head (C,E,G,I) even though each RNAi line reduces levels of the target below detection levels (D,F,H,J, red arrows). (K) Quantitative RT-PCR showing target transcript levels from entire wild type and mutant eye-antennal discs. The remaining transcripts in each mutant sample are from the ventral half of the disc. Note that the expression of *toy*, *tsh*, and *tio* is reduced by approximately 50%, which is expected based on the *DE-GAL4* pattern. *ey* transcripts appear unaffected although we observe a complete elimination of Ey protein in the dorsal half of the eye (F). One possible explanation is that there is compensatory expression of the *ey* gene within the ventral half of the retina. Additionally, there is about 10% increase in *tsh* expression on knockdown of *tio*, and about 32% increase in *tio* expression on knockdown of *tsh*. Note that *so* expression is not affected in any of the mutants, which is expected since the eyes are structurally normal. All qPCR samples were run in biological triplicate and normalized to the reference gene *rp49*. (Scale bars, **50** μm).
(TIF)

S2 Fig Tsh protein is completely removed after 12hrs of RNAi treatment. (A-D) *tub-GAL80*^*ts*^*; DE> UAS-tsh RNAi + UAS-GFP*. Light microscope images of third larval instar eye-antennal discs. Embryos and larvae were raised at 18°C (permissive temp for GAL80) until the third larval instar and then shifted to the non-permissive temperature (30°C). Larvae were dissected and Tsh protein level was monitored in the dorsal compartment at 6hrs (A, bright green arrow), 9hrs (B, dark green arrow), 11hrs (C, orange arrow), and 12hrs (D, red arrow) after RNAi induction. Tsh protein is completely eliminated from the dorsal half of the retina by 12hrs. In [12] we showed that Toy protein is eliminated after 10hrs of RNAi treatment. These temporal windows are incorporated into our calculations for determining the critical windows for Toy/Tsh activity. (Scale bars, **50** μm).
(TIF)

S3 Fig Restoration of Tsh protein takes 38hrs after RNAi induction ceases. (A-F) *tub-GAL80*^*ts*^*; DE> UAS-tsh RNAi + UAS-GFP*. Light microscope images of third larval instar eye-antennal discs. Embryos and larvae were raised at 30°C (non-permissive temp for GAL80) until the third larval instar and then shifted to the permissive temperature (18°C). Larvae were dissected and Tsh protein level was monitored in the dorsal compartment at 6hrs (A, red arrow), 10hrs (B, bright orange arrow), 15hrs (C, dark orange arrow), 24hrs (D, hunter green arrow), 30hrs (E, evergreen green arrow), and 38hrs (F, bright green arrow) after RNAi induction ceased. Tsh protein has recovered to wild type levels by 38hrs. In [12] we demonstrated that Toy protein levels recover 44hrs after RNAi induction has ended. These temporal windows are incorporated into our calculations for determining the critical windows for Toy/Tsh activity. (Scale bars, **50** μm).
(TIF)

S4 Fig Effect of losing either Toy or Tsh individually using *DE-GAL4*, *ey-GAL4*, *eya-GAL4* and *tio-GAL4*. (A) *DE-GAL4*, *UAS-tsh RNAi*, abnormal wing posture that is similar to the *aeroplane-like* (*ae-l*) allele of *tsh*. (B,C) *ey-GAL4*, *UAS-toy RNA*i, normal head structure. (D,E) *ey-GAL4*, *UAS-tsh RNAi;* ventral eye development is inhibited. (F,G) *eya-GAL4*, *UAS-toy RNAi*; normal head structure. (H,I) *tio-GAL4*, *UAS-toy RNAi*; normal head structure. (J,K) *tio-GAL4*, *UAS-tsh RNAi*; normal head development. (L) *tio-GAL4*, *UAS-lacZ* leg disc showing *tio* expression pattern. (M-P) *tio-GAL4*, *UAS-tsh RNAi*. (M) The loss of Tsh leads to the duplication of the leg disc (orange arrows). This is similar to the antennal duplication that results from the loss of Tsh. (N) The resulting adult legs (right) are considerably smaller than their wild type counterparts (left). (O) Some adult legs fail to extend and are often buried within the abdomen, orange arrows. (P) The internalized adult legs after dissection. (Scale bars, **100** μm).
(TIF)

S5 Fig Control of early eye-antennal disc development by Toy/Tsh is independent of the RD network. (A-F) Light microscope images of eye-antennal discs from either *Act5C>y+>GAL4*, *UAS-GFP*, *UAS-toy RNAi*, *UAS-tsh RNAi* (A-E) or *Act5C>y+>GAL4*, *UAS-LacZ*, *UAS-toy RNAi*, *UAS-tsh RNAi* (F) containing clones simultaneously expressing *toy* RNAi and *tsh* RNAi constructs (marked with GFP or LacZ). (A) In clones, the RNAi constructs efficiently knockdown expression of both *toy* and *tsh*. (B-F) The expression of *ey* (B), *eya* (C), *dac* (D), *hth* (E), and *eyg-GFP* (F) are not affected by the combined loss of Toy/Tsh. Anterior is to the right. (Scale bars, **25** μm).
(TIF)

S6 Fig Defects in cell proliferation are not a significant contributor to the headless phenotype of removing Tsh/Toy. (A-C, E-G) In these graphs the density of Hoechst, EdU, and PH3 positive cells in the dorsal and ventral compartments after *toy/tsh* have been knocked-down for 24hrs (A-C) and for 36hrs (E-G) is presented. (A and E) To account for the difference in cell number between dorsal and ventral compartments, a ratio of Hoechst positive cells in dorsal (or ventral) to the total number of cells in the entire disc (D+V) was determined. (B,C,F,G) To accurately determine the percentage of cells in each compartment that express either EdU or PH3, we divided the number of cells expressing these markers within a single compartment by the total number of Hoechst positive cells in the same compartment. Each graph is therefore comparing ratios of EdU and PH3 positive cells within the dorsal and ventral domains of the eye disc. All eye discs were of the following genotype: *tub-GAL80*^*ts*^*; DE-GAL4*, *UAS-toy RNAi*, *UAS-tsh RNAi*. (A,E) The loss of Toy/Tsh results in a dorsal compartment that contains significantly fewer cells than the ventral compartment. (B,C,F,G) However, there are only slight differences in the percentage of cells that are in either S or M phases of the cell cycle. (D-D”‘) Light microscope images of discs that were analyzed for panels A-C. The yellow line demarcates the midline. The orange arrows mark the dorsal compartment where both *toy* and *tsh* expression is knocked-down. (H-H”‘) Light microscope images of discs that were analyzed for panels E-G. The orange arrows mark the dorsal compartment where both *toy* and *tsh* expression is knocked-down. Anterior is to the right. N = 18 in each experiment, *P ≤ 0.*1*, **P ≤ 0.*01*, ***P ≤ 0.*001*, ****P ≤ 0.0001 (Scale bars, **50** μm).
(TIF)

S7 Fig Repression of *cut* does not function through the core RD network. (A-F) Light microscope images of third instar antennal discs. (A) Forced expression of *tsh* induces *eya* expression (orange arrow), induces the formation of an ectopic eye (green arrows). (B,C) The repression of *cut* (red arrow) by *ey* occurs only when an ectopic eye is induced (green arrow). This is different than *tsh*, which can repress *cut* in the absence of ectopic eye formation. (D) Ey cannot repress *cut* expression in the absence of key eye specification factor, So (E,F) Neither *so* nor *eya* appear capable of repressing *cut* within the *dpp-GAL4* ventral expression domain. Posterior is to the right. (Scale bars, **50** μm).
(TIF)

S8 Fig The Zn finger domains of Tsh/Tio function redundantly. (A-G) Light microscope images of third instar antennal discs in which Tsh*/*Tio protein variants are expressed via *dpp*-GAL4. Each panel depicts the effect that removal of an individual zinc finger domain has on the ability of either Tsh or Tio to repress *cut* expression. In all cases, the deletion of an individual zinc finger domain does not impair the ability to repress *cut* within the antenna. This suggests that the zinc fingers either work redundantly or cooperatively to enable Tsh and Tio to bind to their DNA targets. Posterior is to the right. (Scale bars, **50** μm).
(TIF)

S9 Fig (A-L, N-BB) Light microscope images of third instar antennal discs. (M) Light microscope image of an adult antenna and arista. (A) *so*^*1*^ –Ey protein is not found within the antennal disc of *so*^*1*^ mutants. (B,C) Both Tsh (B) and Tio (C) are capable of activating *ey* (green arrows) expression within the ventral antenna. (D) *eya*^*2*^—Ey protein is not found within the antennal disc of *eya*^*2*^ mutants. (E,F) Both Tsh (E) and Tio (F) are capable of activating *ey* (green arrows) expression within the ventral antenna. (G) wildtype—Antennal disc showing normal *Wg reporter* and *cut* expression. (H) Tsh is capable of activating *Wg Reporter* expression within the ventral antenna (I) wild type–*tsh* expression does not induce *Antp* transcription. (J) wild type–*tio* expression does not induce *Antp* transcription. (K) wild type—*cut* expression pattern. (L) *dpp-GAL4*, *UAS-cut RNAi*–*cut* expression is lost in the *dpp* expression domain. (M) *dpp-GAL4*, *UAS-cut RNAi–*the arista is unaffected by the loss of *cut*. (N) *ey*^*LB*^*–hth* expression pattern. (O,O’) *dpp-GAL4 UAS-tsh; ey*^*LB*^–*hth* expression is unaffected within the *dpp* expression domain. (P,P’) *dpp-GAL4 UAS-tio; ey*^*LB*^–*hth* expression is unaffected within the *dpp* expression domain. (Q) *ey*^*LB*^–*Dll* expression pattern. (R,R’) *dpp-GAL4 UAS-tsh; ey*^*LB*^–*Dll* expression is unaffected within the *dpp* expression domain. (S,S’) *dpp-GAL4 UAS-tio; ey*^*LB*^–*Dll* expression is unaffected within the *dpp* expression domain. (T) *ey*^*LB*^–*al* expression pattern. *dpp-GAL4 UAS-tsh; ey*^*LB*^–*al* expression is lost within the head epidermis (orange arrow) but is unaffected within the aristal segment. (U,U’) *dpp-GAL4 UAS-tio; ey*^*LB*^–*al* expression is lost within the head epidermis (orange arrow) but is unaffected within the aristal segment. (W) wild type–*Lim1* expression pattern. (X,X’) *dpp-GAL4 UAS-tsh; ey*^*LB*^–Like *al*, *Lim1* expression is inhibited by the expression of *tsh*. (Y,Y’) *dpp-GAL4 UAS-tio; ey*^*LB*^–Like *al*, *Lim1* expression is inhibited by the expression of *tio*. (Z) *ey*^*LB*^–*dac* and *ss* expression pattern. (AA,AA’) *dpp-GAL4 UAS-tsh; ey*^*LB*^–*dac* expression is induced within the head epidermis by Tsh (green arrow) independent of Ey. A portion of its normal pattern (orange arrow) is lost simultaneously. (BB,BB’) *dpp-GAL4 UAS-tio; ey*^*LB*^–*dac* expression is induced within the head epidermis by Tio (green arrow) independent of Ey. A portion of its normal pattern (orange arrow) is lost simultaneously. Posterior is to the right. (Scale bars, **50** μm).
(TIF)

S10 Fig (A-C) Light microscope images of *dpp-GAL4*, *UAS-tsh* third larval instar eye-antennal discs. (A) In this example, the targeted expression of *tsh* does not induce *ey* expression (14.3%). The adult tissues are slightly disrupted without any homeotic transformations. (B) In some discs (57.1%), the expression of *tsh* induces *ey* expression within the head epidermis (green arrow) which results in the transformation of head epidermis into eye tissue. (C) In some others (28.6%), the expression of *tsh* can activate *ey* (green arrow) and repress both *Lim1* (orange arrow) and *al* (red arrow) expression within the aristal segment. This transforms the arista into a compound eye. Anterior is to the right. N = 21 (Scale bars, **50** μm).
(TIF)

S11 Fig Schematic drawing of the *ey*^*LB*^ allele. The CRISPR/Cas9 system was used to delete an eye-specific enhancer within the eyeless locus.
(TIF)
