# Supplementary figures and images for: Allocation of distinct organ fates from a precursor field requires a shift in expression and function of gene regulatory networks
Source: PLoS Genet. 2018 Jan 19;14(1):e1007185. doi: 10.1371/journal.pgen.1007185 (PMC5792024; doi:10.1371/journal.pgen.1007185)

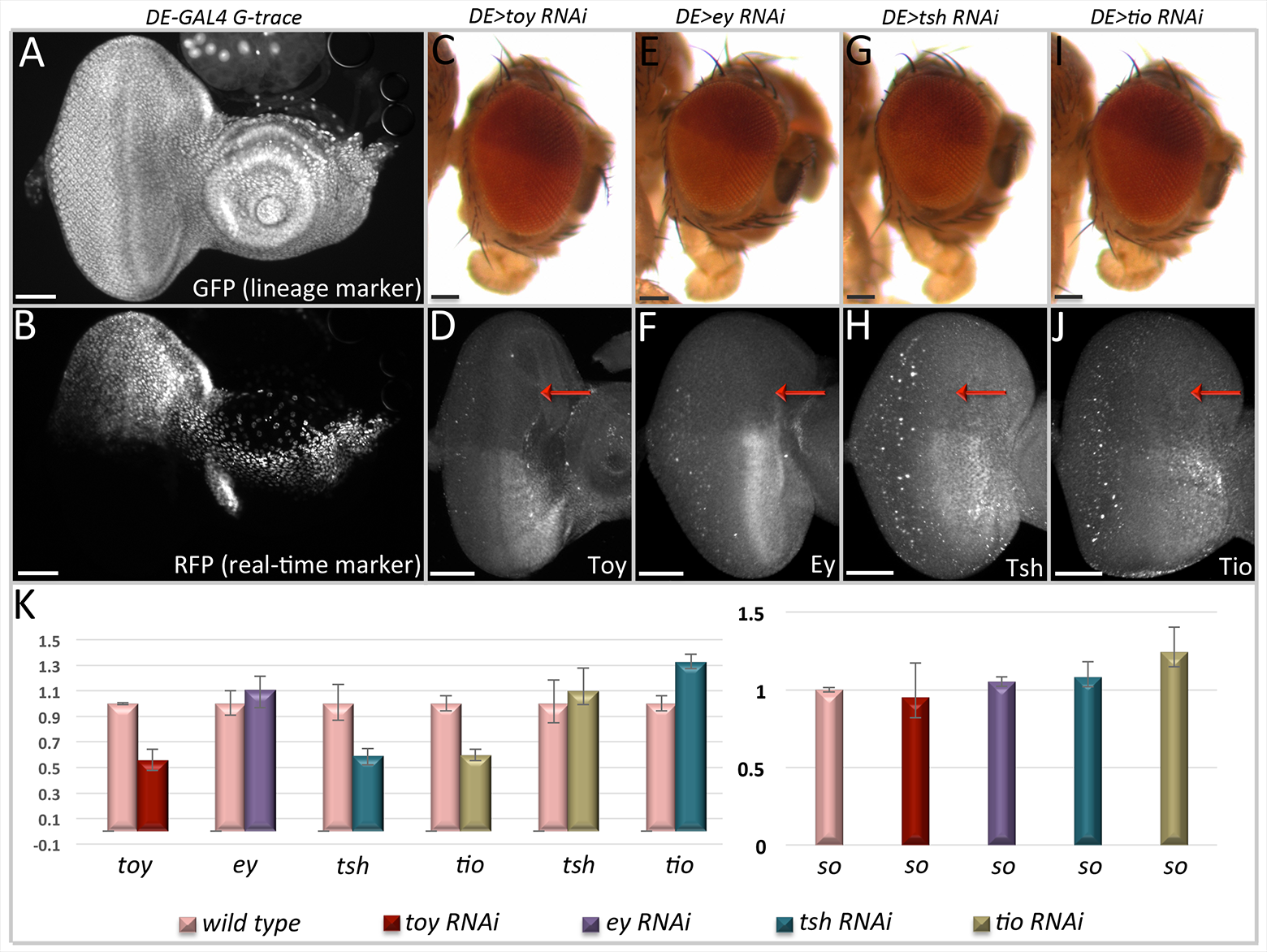

Supplement: S1 Fig — (A,B) Light microscope images showing the lineage tracing (A) and real time (B) expression of the DE-GAL4 driver. Since GFP (lineage marker) is present in all cells of the imaginal disc (A), the driver should be expressed throughout the entire disc at some point in development. In a previous study [12] we confirmed that DE-GAL4 driver is expressed throughout the disc during embryogenesis and the first larval instar. (C,E,G,I) Light microscope images of adult heads. (D,F,H,J) Light microscope images of third instar eye discs. (C,D) DE>toy RNAi (E,F) DE>ey RNAi (G,H) DE>tsh RNAi (I,J) DE>tio RNAi: expression of each RNAi individually has no effect on the overall structure of the adult head (C,E,G,I) even though each RNAi line reduces levels of the target below detection levels (D,F,H,J, red arrows). (K) Quantitative RT-PCR showing target transcript levels from entire wild type and mutant eye-antennal discs. The remaining transcripts in each mutant sample are from the ventral half of the disc. Note that the expression of toy, tsh, and tio is reduced by approximately 50%, which is expected based on the DE-GAL4 pattern. ey transcripts appear unaffected although we observe a complete elimination of Ey protein in the dorsal half of the eye (F). One possible explanation is that there is compensatory expression of the ey gene within the ventral half of the retina. Additionally, there is about 10% increase in tsh expression on knockdown of tio, and about 32% increase in tio expression on knockdown of tsh. Note that so expression is not affected in any of the mutants, which is expected since the eyes are structurally normal. All qPCR samples were run in biological triplicate and normalized to the reference gene rp49. (Scale bars, 50 μm). (TIF) [file pgen.1007185.s001.tif]

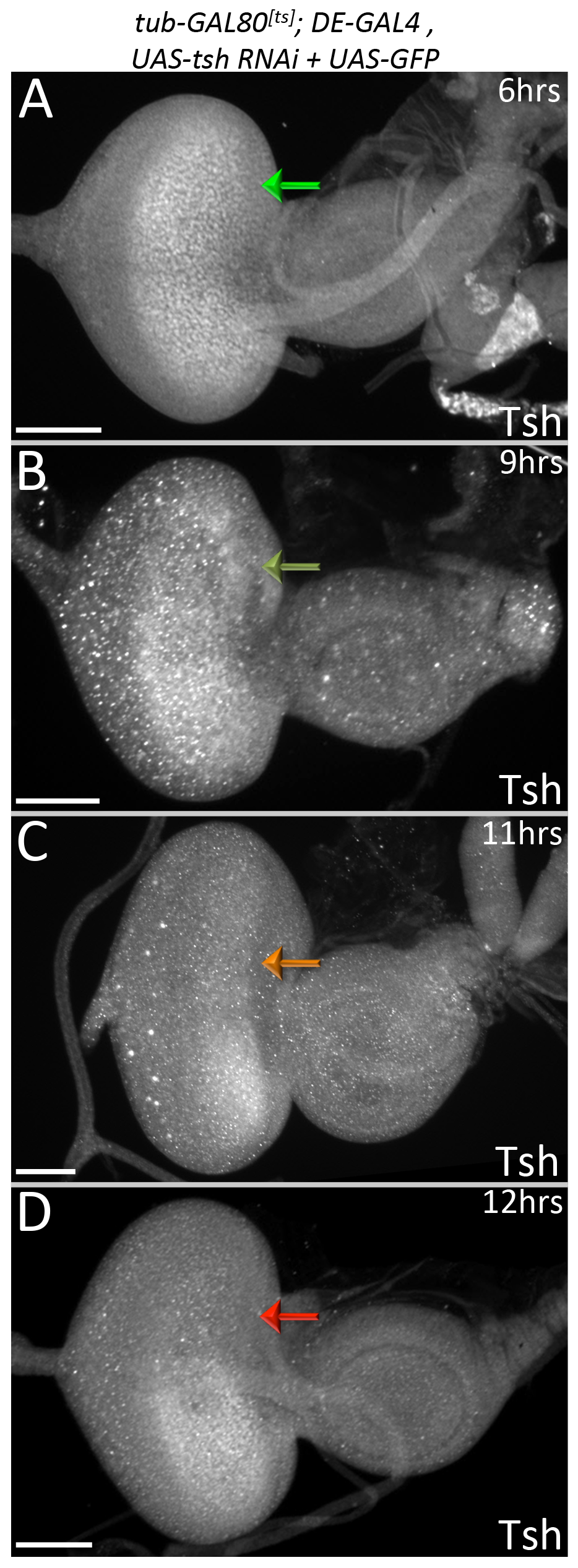

Supplement: S2 Fig — (A-D) tub-GAL80ts; DE> UAS-tsh RNAi + UAS-GFP. Light microscope images of third larval instar eye-antennal discs. Embryos and larvae were raised at 18°C (permissive temp for GAL80) until the third larval instar and then shifted to the non-permissive temperature (30°C). Larvae were dissected and Tsh protein level was monitored in the dorsal compartment at 6hrs (A, bright green arrow), 9hrs (B, dark green arrow), 11hrs (C, orange arrow), and 12hrs (D, red arrow) after RNAi induction. Tsh protein is completely eliminated from the dorsal half of the retina by 12hrs. In [12] we showed that Toy protein is eliminated after 10hrs of RNAi treatment. These temporal windows are incorporated into our calculations for determining the critical windows for Toy/Tsh activity. (Scale bars, 50 μm). (TIF) [file pgen.1007185.s002.tif]

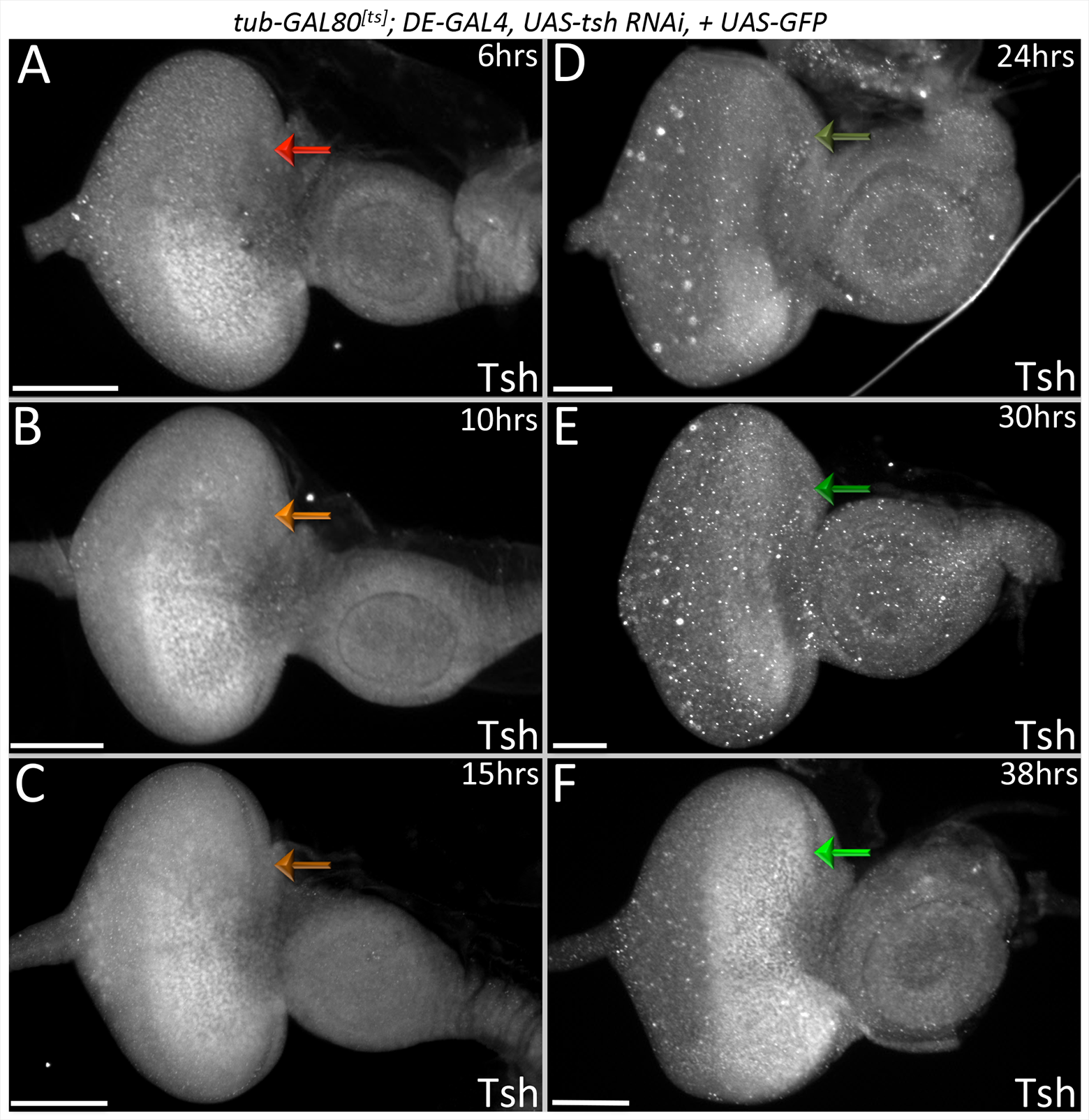

Supplement: S3 Fig — (A-F) tub-GAL80ts; DE> UAS-tsh RNAi + UAS-GFP. Light microscope images of third larval instar eye-antennal discs. Embryos and larvae were raised at 30°C (non-permissive temp for GAL80) until the third larval instar and then shifted to the permissive temperature (18°C). Larvae were dissected and Tsh protein level was monitored in the dorsal compartment at 6hrs (A, red arrow), 10hrs (B, bright orange arrow), 15hrs (C, dark orange arrow), 24hrs (D, hunter green arrow), 30hrs (E, evergreen green arrow), and 38hrs (F, bright green arrow) after RNAi induction ceased. Tsh protein has recovered to wild type levels by 38hrs. In [12] we demonstrated that Toy protein levels recover 44hrs after RNAi induction has ended. These temporal windows are incorporated into our calculations for determining the critical windows for Toy/Tsh activity. (Scale bars, 50 μm). (TIF) [file pgen.1007185.s003.tif]

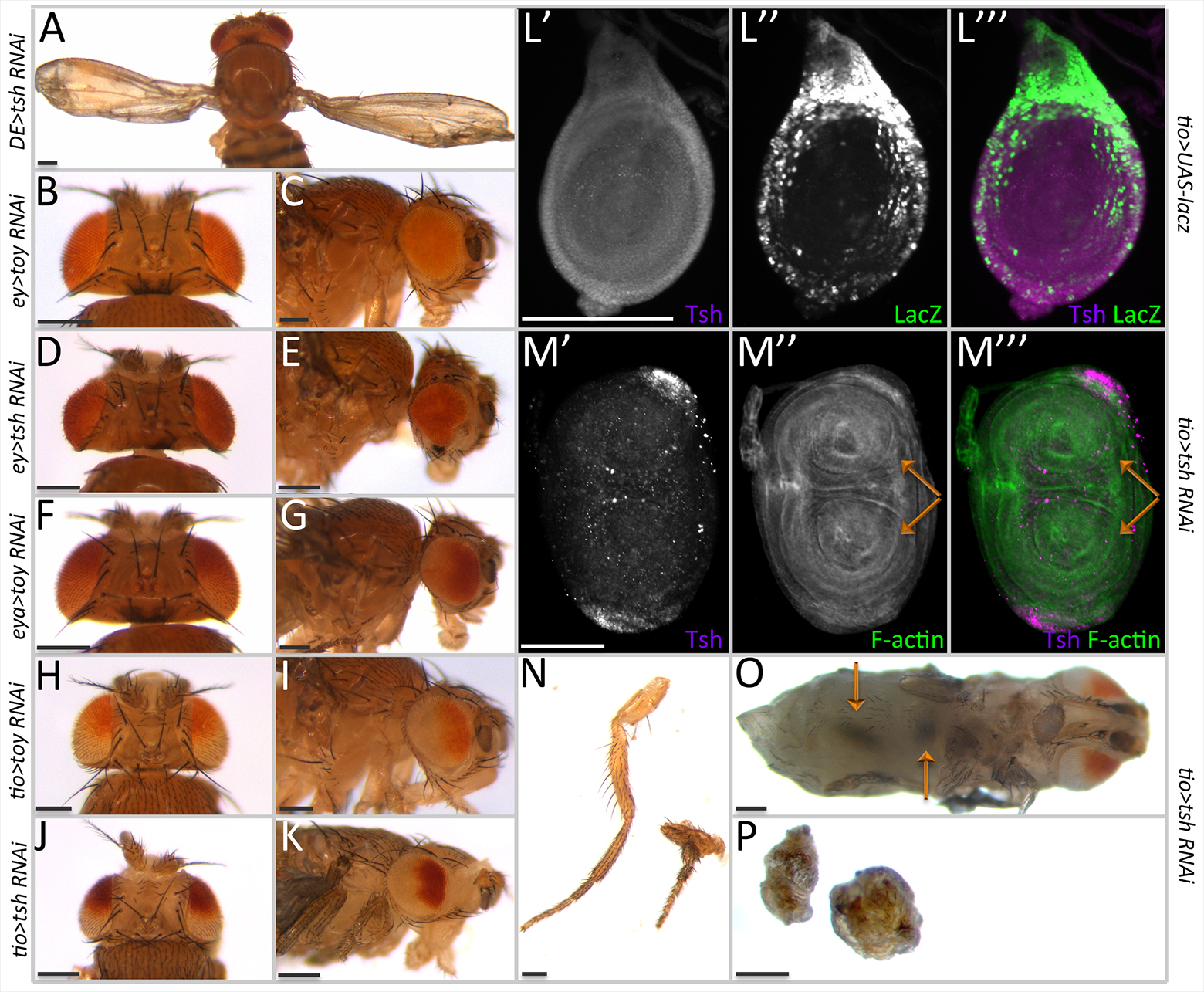

Supplement: S4 Fig — (A) DE-GAL4, UAS-tsh RNAi, abnormal wing posture that is similar to the aeroplane-like (ae-l) allele of tsh. (B,C) ey-GAL4, UAS-toy RNAi, normal head structure. (D,E) ey-GAL4, UAS-tsh RNAi; ventral eye development is inhibited. (F,G) eya-GAL4, UAS-toy RNAi; normal head structure. (H,I) tio-GAL4, UAS-toy RNAi; normal head structure. (J,K) tio-GAL4, UAS-tsh RNAi; normal head development. (L) tio-GAL4, UAS-lacZ leg disc showing tio expression pattern. (M-P) tio-GAL4, UAS-tsh RNAi. (M) The loss of Tsh leads to the duplication of the leg disc (orange arrows). This is similar to the antennal duplication that results from the loss of Tsh. (N) The resulting adult legs (right) are considerably smaller than their wild type counterparts (left). (O) Some adult legs fail to extend and are often buried within the abdomen, orange arrows. (P) The internalized adult legs after dissection. (Scale bars, 100 μm). (TIF) [file pgen.1007185.s004.tif]

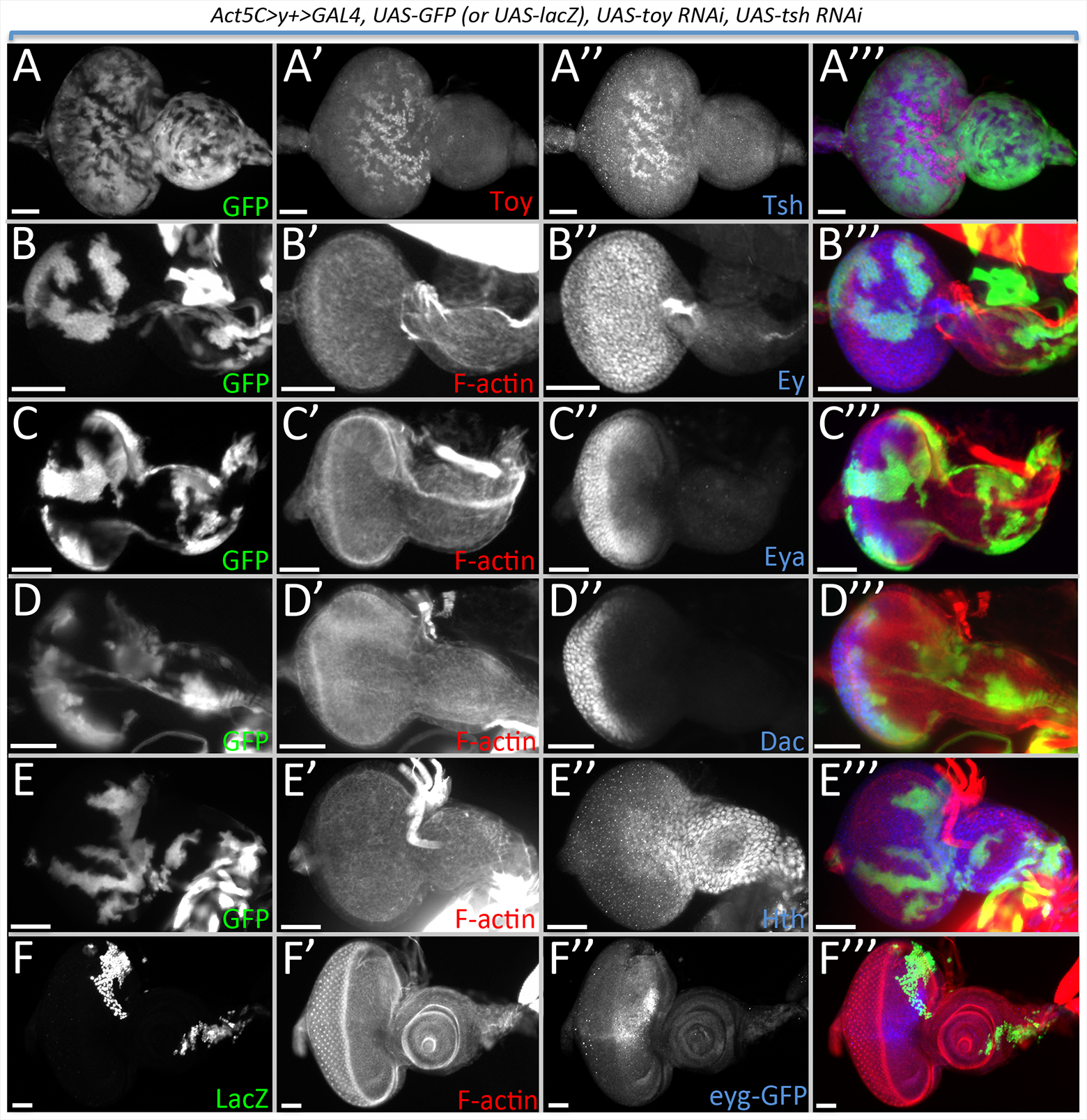

Supplement: S5 Fig — (A-F) Light microscope images of eye-antennal discs from either Act5C>y+>GAL4, UAS-GFP, UAS-toy RNAi, UAS-tsh RNAi (A-E) or Act5C>y+>GAL4, UAS-LacZ, UAS-toy RNAi, UAS-tsh RNAi (F) containing clones simultaneously expressing toy RNAi and tsh RNAi constructs (marked with GFP or LacZ). (A) In clones, the RNAi constructs efficiently knockdown expression of both toy and tsh. (B-F) The expression of ey (B), eya (C), dac (D), hth (E), and eyg-GFP (F) are not affected by the combined loss of Toy/Tsh. Anterior is to the right. (Scale bars, 25 μm). (TIF) [file pgen.1007185.s005.tif]

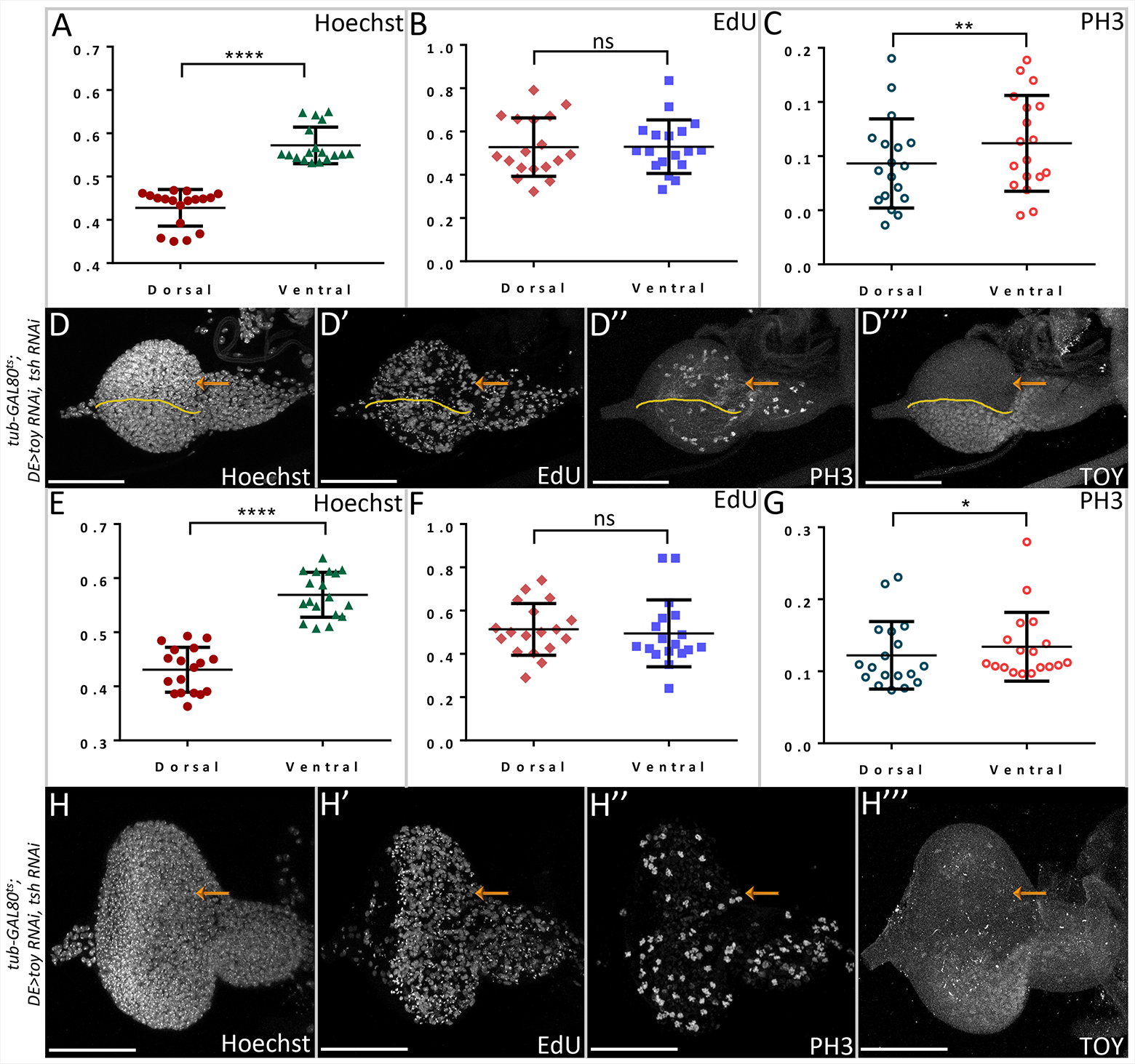

Supplement: S6 Fig — (A-C, E-G) In these graphs the density of Hoechst, EdU, and PH3 positive cells in the dorsal and ventral compartments after toy/tsh have been knocked-down for 24hrs (A-C) and for 36hrs (E-G) is presented. (A and E) To account for the difference in cell number between dorsal and ventral compartments, a ratio of Hoechst positive cells in dorsal (or ventral) to the total number of cells in the entire disc (D+V) was determined. (B,C,F,G) To accurately determine the percentage of cells in each compartment that express either EdU or PH3, we divided the number of cells expressing these markers within a single compartment by the total number of Hoechst positive cells in the same compartment. Each graph is therefore comparing ratios of EdU and PH3 positive cells within the dorsal and ventral domains of the eye disc. All eye discs were of the following genotype: tub-GAL80ts; DE-GAL4, UAS-toy RNAi, UAS-tsh RNAi. (A,E) The loss of Toy/Tsh results in a dorsal compartment that contains significantly fewer cells than the ventral compartment. (B,C,F,G) However, there are only slight differences in the percentage of cells that are in either S or M phases of the cell cycle. (D-D”‘) Light microscope images of discs that were analyzed for panels A-C. The yellow line demarcates the midline. The orange arrows mark the dorsal compartment where both toy and tsh expression is knocked-down. (H-H”‘) Light microscope images of discs that were analyzed for panels E-G. The orange arrows mark the dorsal compartment where both toy and tsh expression is knocked-down. Anterior is to the right. N = 18 in each experiment, *P ≤ 0.1, **P ≤ 0.01, ***P ≤ 0.001, ****P ≤ 0.0001 (Scale bars, 50 μm). (TIF) [file pgen.1007185.s006.tif]

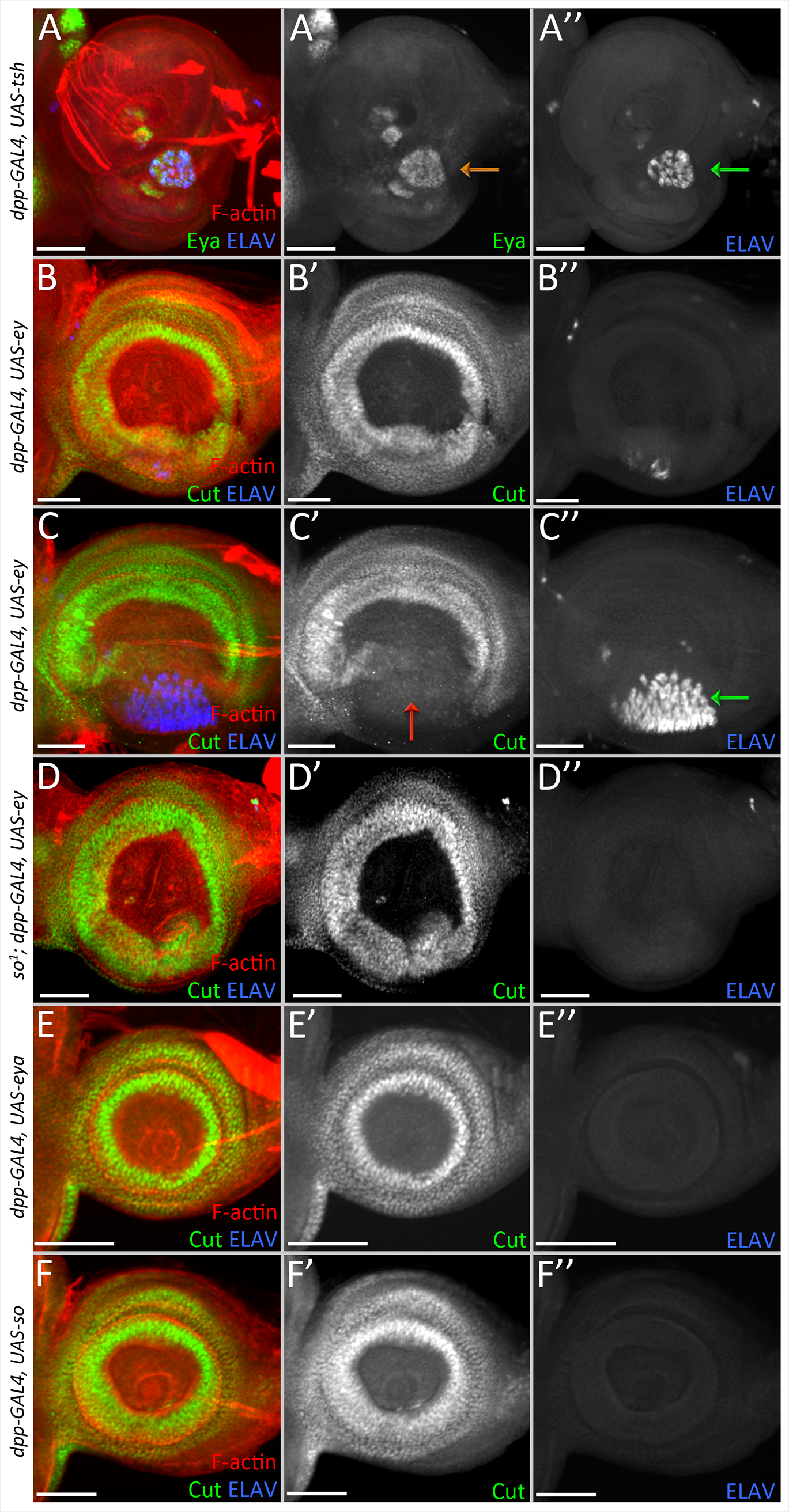

Supplement: S7 Fig — (A-F) Light microscope images of third instar antennal discs. (A) Forced expression of tsh induces eya expression (orange arrow), induces the formation of an ectopic eye (green arrows). (B,C) The repression of cut (red arrow) by ey occurs only when an ectopic eye is induced (green arrow). This is different than tsh, which can repress cut in the absence of ectopic eye formation. (D) Ey cannot repress cut expression in the absence of key eye specification factor, So (E,F) Neither so nor eya appear capable of repressing cut within the dpp-GAL4 ventral expression domain. Posterior is to the right. (Scale bars, 50 μm). (TIF) [file pgen.1007185.s007.tif]

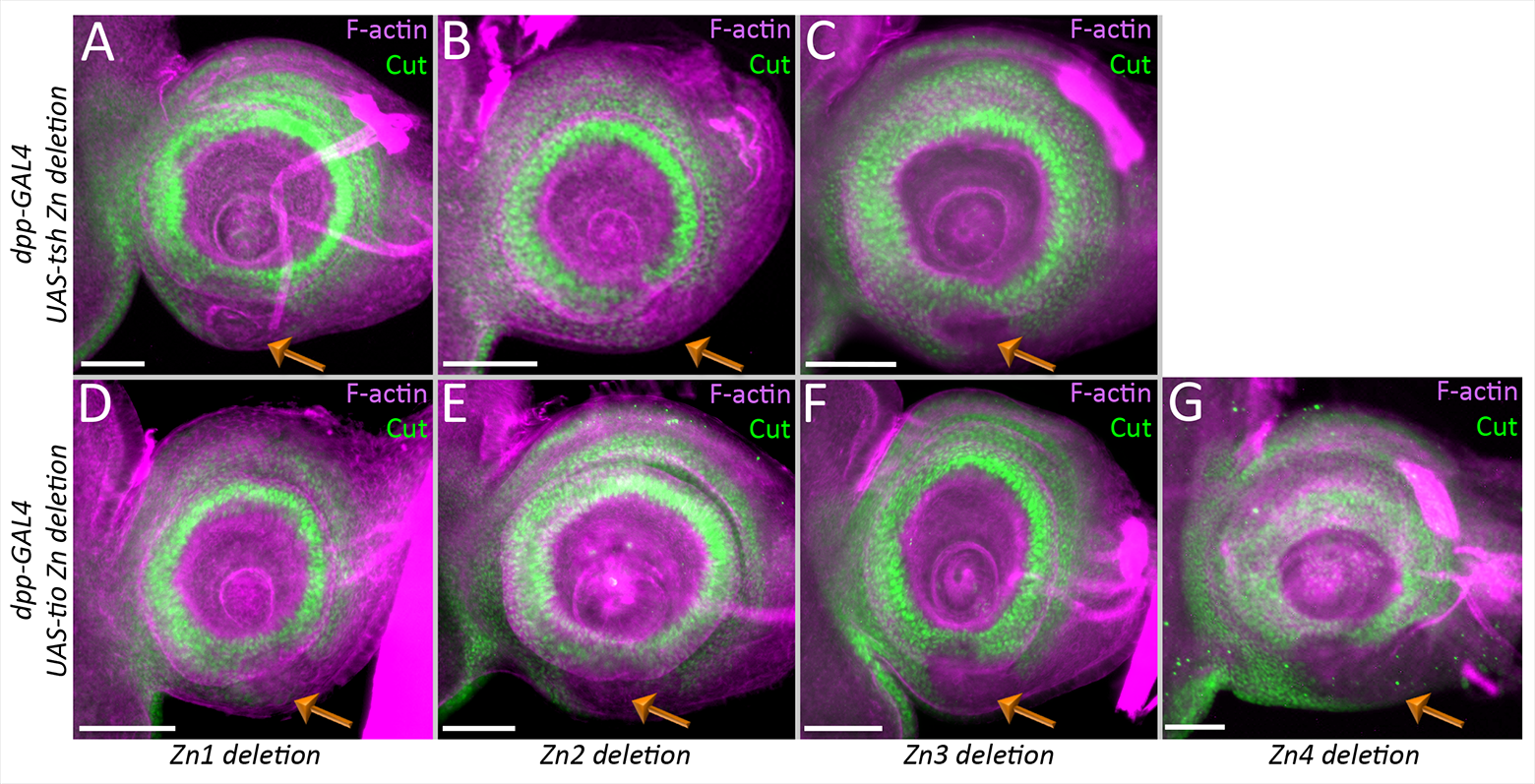

Supplement: S8 Fig — (A-G) Light microscope images of third instar antennal discs in which Tsh/Tio protein variants are expressed via dpp-GAL4. Each panel depicts the effect that removal of an individual zinc finger domain has on the ability of either Tsh or Tio to repress cut expression. In all cases, the deletion of an individual zinc finger domain does not impair the ability to repress cut within the antenna. This suggests that the zinc fingers either work redundantly or cooperatively to enable Tsh and Tio to bind to their DNA targets. Posterior is to the right. (Scale bars, 50 μm). (TIF) [file pgen.1007185.s008.tif]

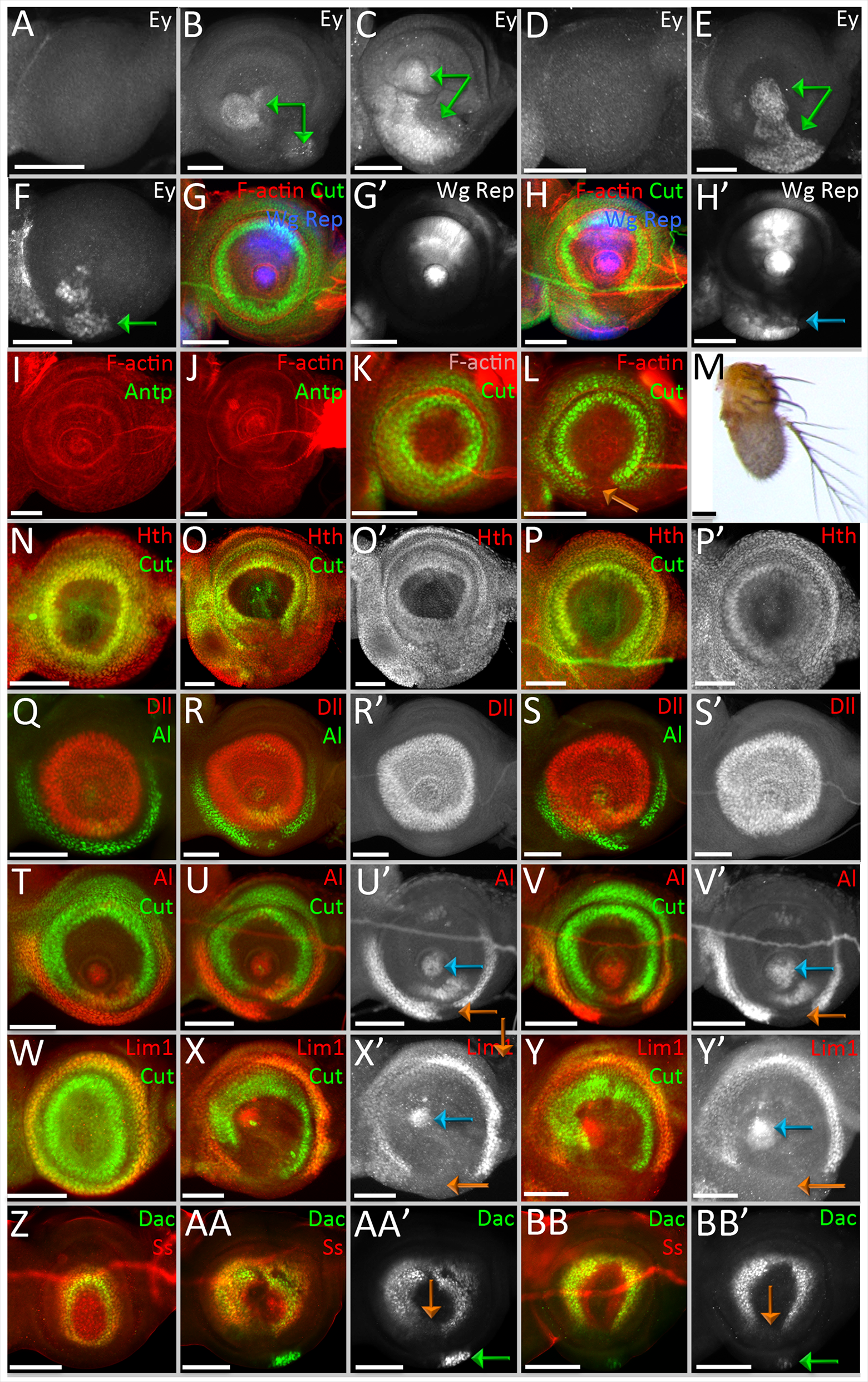

Supplement: S9 Fig — (A-L, N-BB) Light microscope images of third instar antennal discs. (M) Light microscope image of an adult antenna and arista. (A) so1 –Ey protein is not found within the antennal disc of so1 mutants. (B,C) Both Tsh (B) and Tio (C) are capable of activating ey (green arrows) expression within the ventral antenna. (D) eya2—Ey protein is not found within the antennal disc of eya2 mutants. (E,F) Both Tsh (E) and Tio (F) are capable of activating ey (green arrows) expression within the ventral antenna. (G) wildtype—Antennal disc showing normal Wg reporter and cut expression. (H) Tsh is capable of activating Wg Reporter expression within the ventral antenna (I) wild type–tsh expression does not induce Antp transcription. (J) wild type–tio expression does not induce Antp transcription. (K) wild type—cut expression pattern. (L) dpp-GAL4, UAS-cut RNAi–cut expression is lost in the dpp expression domain. (M) dpp-GAL4, UAS-cut RNAi–the arista is unaffected by the loss of cut. (N) eyLB–hth expression pattern. (O,O’) dpp-GAL4 UAS-tsh; eyLB–hth expression is unaffected within the dpp expression domain. (P,P’) dpp-GAL4 UAS-tio; eyLB–hth expression is unaffected within the dpp expression domain. (Q) eyLB–Dll expression pattern. (R,R’) dpp-GAL4 UAS-tsh; eyLB–Dll expression is unaffected within the dpp expression domain. (S,S’) dpp-GAL4 UAS-tio; eyLB–Dll expression is unaffected within the dpp expression domain. (T) eyLB–al expression pattern. dpp-GAL4 UAS-tsh; eyLB–al expression is lost within the head epidermis (orange arrow) but is unaffected within the aristal segment. (U,U’) dpp-GAL4 UAS-tio; eyLB–al expression is lost within the head epidermis (orange arrow) but is unaffected within the aristal segment. (W) wild type–Lim1 expression pattern. (X,X’) dpp-GAL4 UAS-tsh; eyLB–Like al, Lim1 expression is inhibited by the expression of tsh. (Y,Y’) dpp-GAL4 UAS-tio; eyLB–Like al, Lim1 expression is inhibited by the expression of tio. (Z) eyLB–dac and ss expression pattern. (AA,AA’) dp [file pgen.1007185.s009.tif]

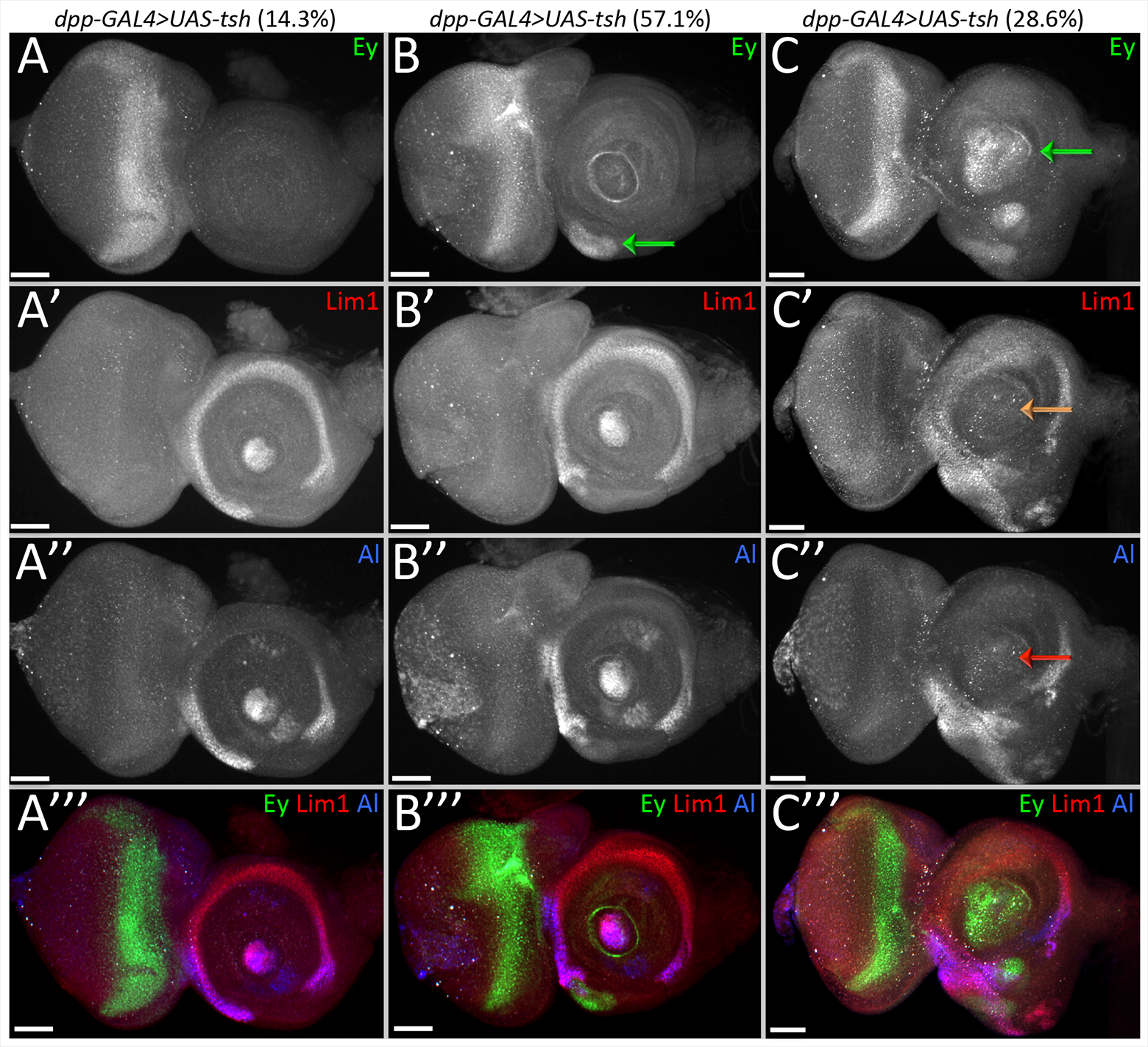

Supplement: S10 Fig — (A-C) Light microscope images of dpp-GAL4, UAS-tsh third larval instar eye-antennal discs. (A) In this example, the targeted expression of tsh does not induce ey expression (14.3%). The adult tissues are slightly disrupted without any homeotic transformations. (B) In some discs (57.1%), the expression of tsh induces ey expression within the head epidermis (green arrow) which results in the transformation of head epidermis into eye tissue. (C) In some others (28.6%), the expression of tsh can activate ey (green arrow) and repress both Lim1 (orange arrow) and al (red arrow) expression within the aristal segment. This transforms the arista into a compound eye. Anterior is to the right. N = 21 (Scale bars, 50 μm). (TIF) [file pgen.1007185.s010.tif]

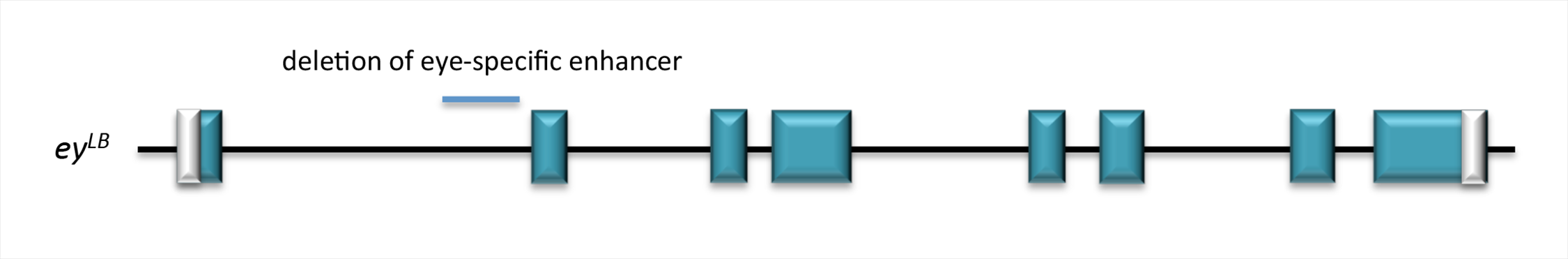

Supplement: S11 Fig — The CRISPR/Cas9 system was used to delete an eye-specific enhancer within the eyeless locus. (TIF) [file pgen.1007185.s011.tif]
